# PRMT1 promotes Warburg effect by regulating the PKM2/PKM1 ratio in non-small cell lung cancer

**DOI:** 10.1038/s41419-024-06898-x

**Published:** 2024-07-15

**Authors:** Lu Peng, Yujiao Zhao, Jiang Tan, Jingyao Hou, Xin Jin, Dong-Xu Liu, Baiqu Huang, Jun Lu

**Affiliations:** 1 The Key Laboratory of Molecular Epigenetics of Ministry of Education (MOE), Northeast Normal University, Changchun, 130024 China; 2 The Institute of Genetics and Cytology, Northeast Normal University, Changchun, 130024 China; 3 Institute of Translational Medicine of Breast Disease Prevention and Treatment, The Second Hospital of Shandong University, Jinan, 250033 China

**Keywords:** Non-small-cell lung cancer, Cancer metabolism

## Abstract

Abnormal epigenetic modifications are involved in the regulation of Warburg effect in tumor cells. Protein arginine methyltransferases (PRMTs) mediate arginine methylation and have critical functions in cellular responses. PRMTs are deregulated in a variety of cancers, but their precise roles in Warburg effect in cancer is largely unknown. Experiments from the current study showed that PRMT1 was highly expressed under conditions of glucose sufficiency. PRMT1 induced an increase in the PKM2/PKM1 ratio through upregulation of PTBP1, in turn, promoting aerobic glycolysis in non-small cell lung cancer (NSCLC). The PRMT1 level in p53-deficient and p53-mutated NSCLC remained relatively unchanged while the expression was reduced in p53 wild-type NSCLC under conditions of glucose insufficiency. Notably, p53 activation under glucose-deficient conditions could suppress USP7 and further accelerate the polyubiquitin-dependent degradation of PRMT1. Melatonin, a hormone that inhibits glucose intake, markedly suppressed cell proliferation of p53 wild-type NSCLC, while a combination of melatonin and the USP7 inhibitor P5091 enhanced the anticancer activity in p53-deficient NSCLC. Our collective findings support a role of PRMT1 in the regulation of Warburg effect in NSCLC. Moreover, combination treatment with melatonin and the USP7 inhibitor showed good efficacy, providing a rationale for the development of PRMT1-based therapy to improve p53-deficient NSCLC outcomes.

## Introduction

According to the latest global data, lung cancer ranks second as a cause of cancer-related deaths worldwide [1]. Non-small cell lung cancer (NSCLC) is the main type of lung cancer, accounting for 85% of all cases, with relatively poor prognosis and treatment effects associated with low survival rates. Cancer cells can reprogram their glucose metabolism by limiting their energy mode predominantly to aerobic glycolysis even under normoxic conditions, a characteristic known as the Warburg effect. Abnormal expression of glucose-metabolizing enzymes, including pyruvate kinase (PK), is crucial for occurrence of the Warburg effect [2, 3]. PKM1 and PKM2 are mutually exclusive products formed by alternative splicing of precursor mRNA encoded by the PK gene, *PKM*. Switching of PKM2 to PKM1 reverses aerobic glycolysis to oxidative phosphorylation. The Warburg effect provides cancer cells advantages in terms of accelerated cell proliferation and avoidance of apoptosis [4, 5]. Thus, improved understanding of the mechanistic links between the Warburg effect and survival control is of paramount significance for the development of novel therapeutics for NSCLC.

PRMT1, the first identified arginine methyltransferase, plays a crucial role in a range of biological processes, including the cell cycle, DNA damage, transcription and signal transduction [6]. Recent studies have demonstrated a regulatory role of PRMT1 in cellular metabolic pathways, including serine metabolism [7], lipid metabolism [8, 9] and glucose homeostasis [10–12]. In addition, high PRMT1 expression is known to contribute to cell proliferation in multiple tumors [13–15]. However, the potential role of PRMT1 in Warburg effect of cancer cells remains largely unknown. Transcriptional and post-transcriptional modifications that regulate PRMT1 stability have been reported [16, 17]. Preliminary evidence has shown that PRMT1 can be degraded by proteasomes [18–20]. However, the mechanistic details of PRMT1 dysregulation in cancers are yet to be established.

Wild-type p53 is a tumor suppressor that regulates multiple physiological functions and its mutation or deficiency commonly contributes to malignant tumor progression. In recent years, a regulatory role of wild-type p53 on glucose metabolism has been uncovered. Wild-type p53 not only inhibits glucose uptake [21, 22] but also regulates the gene expression of proteins related to glucose metabolism [23, 24]. Several studies have demonstrated that p53 is activated under conditions of insufficient nutrient intake and involved in the metabolic regulation of tumor cells [25–27]. Knockdown of PRMT1 is reported to promote p53 stabilization, leading to inhibition of epicardial epithelial-to-mesenchymal transition (EMT) and invasion [28]. PRMT1 has been shown to directly bind p53 and inhibit its transcriptional activity, consequently suppressing cell growth arrest and senescence [29]. However, it remains to be established whether p53, a glucose sensor, is able to regulate Warburg effect via modulation of PRMT1.

Data from the current study showed that activation of p53 mediates PRMT1 ubiquitination-dependent degradation via inhibition of USP7 in response to glucose insufficiency in p53 wild-type NSCLC cells. PRMT1 serves as a glucose-inducible protein that promotes aerobic glycolysis via alternative splicing (AS) of *PKM* and NSCLC cell proliferation. Combination treatment with a USP7 inhibitor and melatonin offers a potential selective therapeutic strategy for p53-deficient NSCLC.

## Results

### PRMT1 responds to high glucose to promote NSCLC cell proliferation

**Fig. 1:**
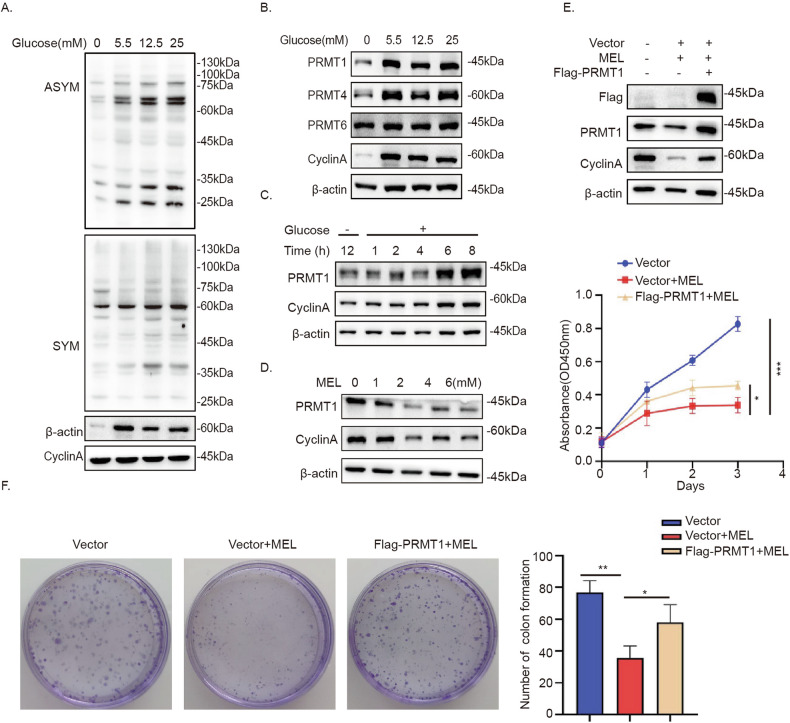
PRMT1 responds to high glucose to promote NSCLC cell proliferation. **A** A549 cells were cultured in medium containing glucose at the indicated concentrations for 18 h and cell lysates subjected to immunoblot analysis with asymmetric arginine methylation (ASYM) or symmetric arginine methylation (SYM) antibodies. **B** A549 cells were cultured in medium containing glucose at the indicated concentrations for 18 h and lysates subjected to immunoblot analysis with PRMT1, PRMT4, or PRMT6 antibodies. Cyclin A was used as the control. **C** A549 cells were glucose-starved for 12 h, followed by stimulation with glucose (25 mM) for the indicated times, and lysates subjected to immunoblot analysis. **D** A549 cells were treated with melatonin at different concentrations for 24 h. **E** A549 cells overexpressing PRMT1 were stimulated with melatonin and proliferation rates measured via CCK-8 assays. **F** Proliferation of A549 cells overexpressing PRMT1 and treated with melatonin was evaluated using the monoclonal assay. Error bars, mean ± SD. ns (non-significant), *P* > 0.05; *P < 0.05; **P < 0.01; ***P < 0.001.

To explore the relationship between arginine methylation modification and Warburg effect in NSCLC, we assessed asymmetric arginine methylation (ASYM) and symmetric arginine methylation (SYM) in A549 cells supplemented with different concentrations of glucose. The results showed significant upregulation of ASYM of proteins from whole cell lysates under increasing glucose concentrations but no significant changes in SYM (Fig. 1A). Notably, protein levels of PRMT1 and PRMT4 were correlated with the glucose concentrations (Fig. 1B). Previous studies clearly indicate that PRMT4 (CARM1) is related to glucose metabolism [30–32] whereas the relationship between PRMT1 and glucose is unclear. Upon reintroduction of glucose into glucose-deprived cells, the PRMT1 level was markedly increased in A549 cells (Fig. 1C). Earlier studies have identified melatonin as an inhibitor of glucose uptake in tumor cells [33, 34]. Here, we observed downregulation of PRMT1 following melatonin treatment in A549 cells (Fig. 1D). Furthermore, overexpression of PRMT1 partially reversed the anti-proliferative effects of melatonin, as determined by CCK-8 viability (Fig. 1E) and colony formation (Fig. 1F) assays. Our results suggest that PRMT1 is induced by high glucose and promotes proliferation of A549 cells.

### PRMT1 promotes the Warburg effect in NSCLC

**Fig. 2:**
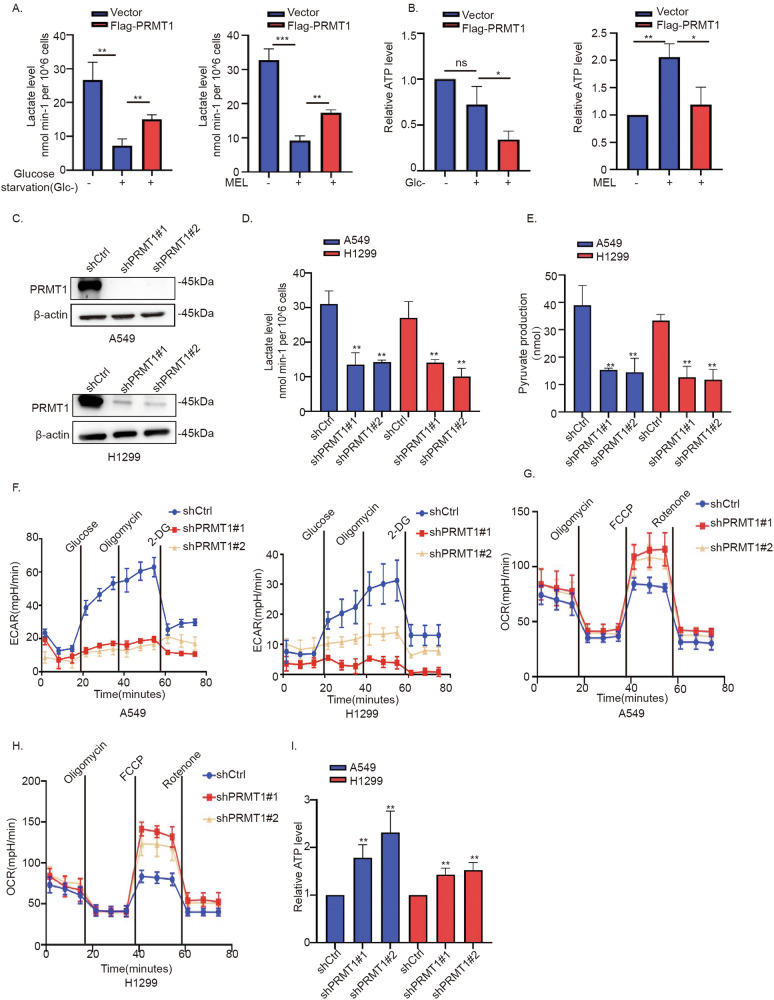
PRMT1 promotes the Warburg effect in NSCLC cells. **A** Alterations in lactate production in A549 cells transfected with the vector and Flag-PRMT1 constructs under glucose starvation and melatonin treatment conditions. **B** Alterations in ATP levels in A549 cells transfected with the vector and Flag-PRMT1 constructs under the condition of glucose insufficiency. **C** Western blot analysis of PRMT1 in A549 and H1299 cells. **D**, **E** Analysis of alterations in lactate and pyruvate production among A549 and H1299 cells transfected with the shCtrl, shPRMT1#1, or shPRMT1#2 constructs. **F** Assessment of the extracellular acidification rate in A549 and H1299 cells transfected with the shCtrl, shPRMT1#1, or shPRMT1#2 constructs. **G**, **H** The OCR curves of A549 cells and H1299 cells infected with shPRMT#1 and shPRMT1#2 plasmids. **I** ATP levels of A549 and H1299 cells with knockdown of PRMT1. Error bars, mean ± SD. ns (non-significant), *P* > 0.05; *P < 0.05; **P < 0.01; ***P < 0.001.

Earlier studies have shown that melatonin suppresses the Warburg effect. Since downregulation of PRMT1 following melatonin treatment in A549 cells, we hypothesized that melatonin inhibited aerobic glycolysis through downregulating PRMT1. To test it, we restored PRMT1 expression under conditions of glucose insufficiency, which resulted in a significant increase in lactate (Fig. 2A) and a concomitant decrease in ATP levels (Fig. 2B). Then we further investigated the influence of PRMT1 on the Warburg effect in NSCLC cells. Upon knockdown of PRMT1 in A549 and H1299 cells, we observed significantly lower lactate and pyruvate production (Fig. 2C–E), along with reduced extracellular acidification rates (ECAR) (Fig. 2F) and increased oxygen consumption rate (OCR) (Fig. 2G, H). In addition, knockdown of PRMT1 elevated the level of ATP produced by OXPHOS (Fig. 2I). The collective results clearly indicate that PRMT1 promotes the Warburg effect in NSCLC cells.

### PRMT1 mediates the splicing of PKM through regulation of PTBP1

**Fig. 3:**
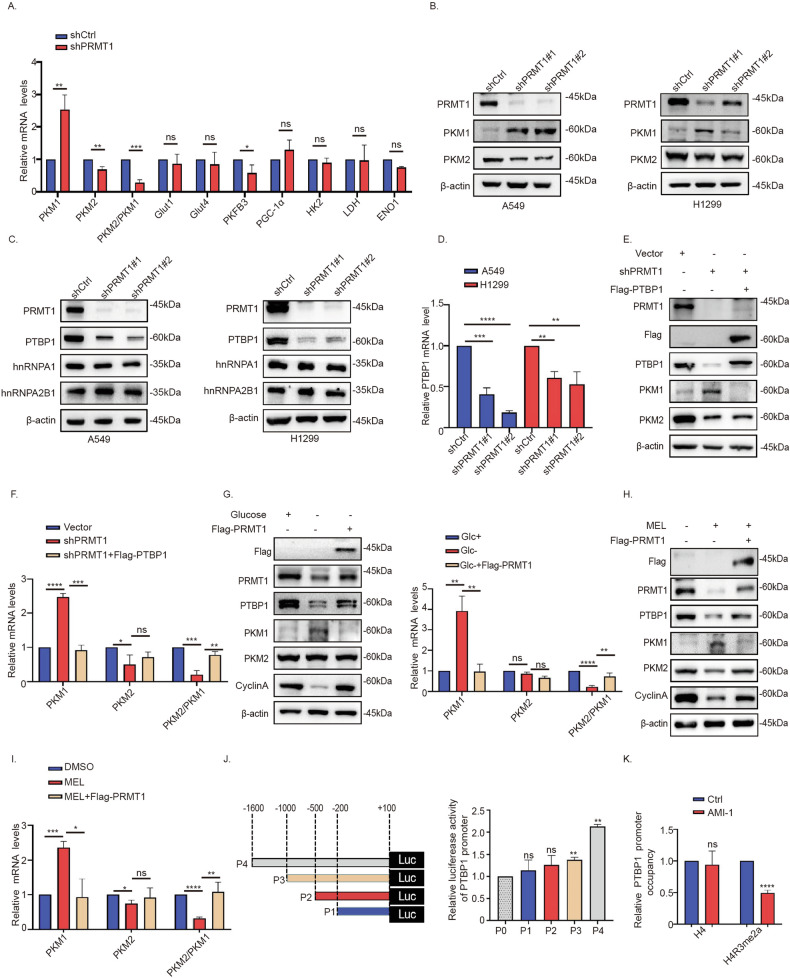
PRMT1 mediates the splicing of PKM through modulation of PTBP1. **A** qPCR analysis of genes encoding glucose metabolism and OXPHOS-related enzymes in A549 cells transfected with the shCtrl and shPRMT1#1 constructs. **B** Western blot analysis of PKM1 and PKM2 in A549 and H1299 cells with PRMT1 knockdown. **C**, **D** Western blot and qPCR analyses of PTBP1, hnRNPA1, and hnRNPA2B1 in A549 and H1299 cells with PRMT1 knockdown. **E**, **F** Western blot and qPCR analyses of PKM1 and PKM2 in A549 cells transfected with the vector, shPRMT1#1, and shPRMT1#2+ Flag-PTBP1 constructs. **G**–**I** Western blot and qPCR analyses of PTBP1, PKM1, and PKM2 in A549-vector and A549-Flag-PRMT1 cells under the condition of glucose insufficiency. **J** PTBP1 promoter fragments of different sizes were constructed and the interactions between PRMT1 and PTBP1 promoter detected via the dual luciferase reporter gene assay. **K** A549 cells were treated with AMI-1 (50 μM). ChIP assays were performed using the anti-H4R3asym2 antibody and immunoprecipitated DNA analyzed via qPCR using PTBP1-specific primers. Error bars, mean ± SD. ns (non-significant), *P* > 0.05; *P < 0.05; **P < 0.01; ***P < 0.001; ****P < 0.0001.

Next, we investigated the mechanisms by which PRMT1 promotes the Warburg effect in NSCLC cells. Knockdown of PRMT1 caused a significant decrease in the PKM2/PKM1 mRNA ratio in A549 and H1299 cells (Fig. 3A, B) and upregulated the PKM1 isoform level (Fig. S1). Splicing of the pyruvate kinase PKM is reported to be mediated by PTBP1, hnRNPA1, and hnRNPA2 [35, 36]. Knockdown of PRMT1 resulted in significant a decrease in both mRNA and protein levels of PTBP1 in A549 and H1299 cells, but not hnRNPA1 or hnRNPA2B1 (Fig. 3C, D). Overexpression of PTBP1 enhanced the inhibitory effect of PRMT1 knockdown on the PKM2/PKM1 ratio in NSCLC cells (Fig. 3E, F). Our results showed that glucose promotes PTBP1 expression and the PKM2/PKM1 ratio in A549 cells (Fig. S2). Restoration of PRMT1 attenuated the inhibitory effect of glucose deficiency on PTBP1 expression and PKM2/PKM1 ratio (Fig. 3G–I). Accordingly, we proposed that PRMT1 increased the PKM2/PKM1 ratio through augmenting expression of PTBP1 under high glucose in NSCLC.

Next, we focused on the mechanism by which PRMT1 regulates PTBP1 expression. Our experiments showed that PRMT1 activates PTBP1 promoter activity in a dose-dependent manner (Fig. S3D). Dual luciferase reporter gene assays further showed that the region from base pair (bp) −1000 to −1600 of PTBP1 promoter is critical (Fig. 3J). Data from chromatin immunoprecipitation (ChIP) experiments revealed that AMI-1, a small-molecule inhibitor of PRMT1, induced a marked decrease in H4R3me2a on the PTBP1 promoter (Fig. 3K, Fig. S3E). These results indicate that PRMT1 promotes PTBP1 transcription through increasing H4R3me2a on the PTBP1 promoter region in NSCLC cells.

### p53 mediates degradation of PRMT1 protein under glucose insufficiency

**Fig. 4:**
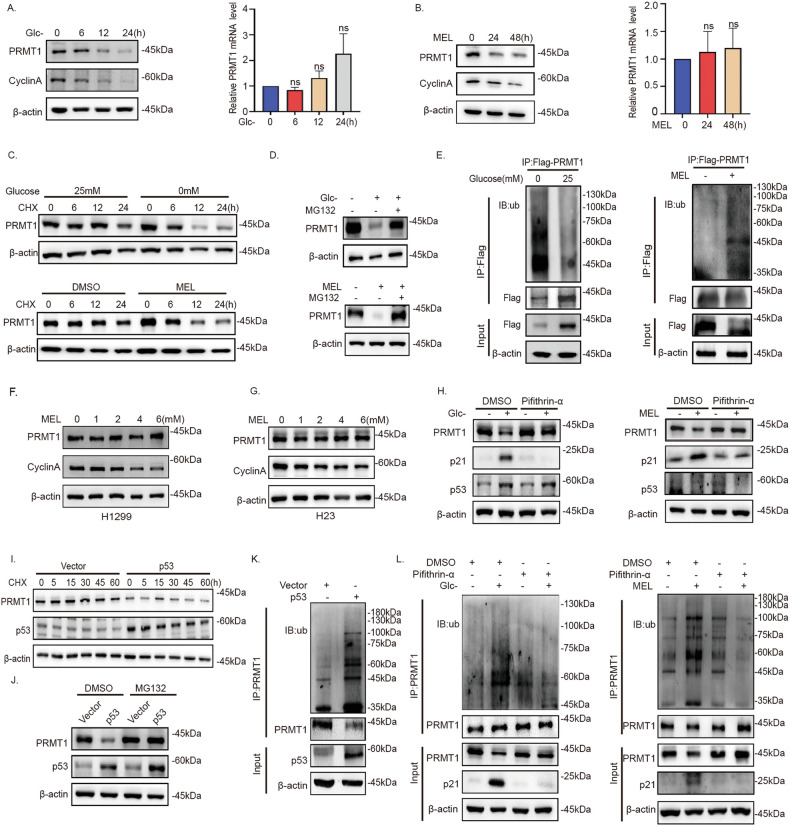
p53 mediates degradation of PRMT1 protein under conditions of glucose insufficiency. **A** Western blot and qPCR analyses of PRMT1 in lysates of A549 cells cultured in 0 mM glucose for 0, 6, 12, and 24 h. **B** Western blot and qPCR analyses of PRMT1 expression in A549 cells treated with melatonin for 24 or 48 h. **C** A549 cells were treated with cycloheximide (100 μg/mL) for the indicated time periods under glucose starvation and melatonin treatment. **D** Western blot analysis of PRMT1 in A549 cells under glucose starvation and treatment with melatonin or MG132 (10 μM). **E** Western blot analysis of PRMT1 ubiquitination after immunoprecipitation with PRMT1 antibody in A549 cells under glucose starvation and melatonin treatment. **F**, **G** Western blot analysis of PRMT1 in p53-deficient H1299 cells and p53-mutated H23 cells treated with different concentrations of melatonin for 24 h. **H** Western blot analysis of PRMT1 in A549 cells treated with pifithrin-α under glucose starvation conditions or melatonin treatment. **I**, **J** Western blot analysis of PRMT1 in A549-Vector and A549-p53 cells treated with CHX (100 μg/mL) or MG132 (10 μM). **K** Western blot analysis of PRMT1 ubiquitination in A549-Vector and A549-p53 cells after immunoprecipitation with PRMT1 antibody. **L** Western blot analysis of PRMT1 ubiquitination after immunoprecipitation with PRMT1 antibody in A549 cells treated with pifithrin-α under glucose starvation conditions and melatonin treatment.

Having confirmed the decrease in PRMT1 during glucose insufficiency, we further explored the mechanisms underlying this phenomenon. Glucose fluctuation had no significant effect on PRMT1 mRNA levels (Fig. 4A, B). One explanation for this observation is that glucose affects the stability of PRMT1 protein. To substantiate this assumption, A549 cells were treated with the protein synthesis inhibitor cycloheximide (CHX) or the proteasome inhibitor MG132. The results indicated that glucose insufficiency promoted the degradation of PRMT1 protein (Fig. 4C, D). Glucose insufficiency induced robust ubiquitylation of PRMT1 (Fig. 4E), indicating PRMT1 degradation mediated by the proteasome pathway under these conditions.

Notably, glucose insufficiency had no effect on PRMT1 protein in p53-deficient H1299 cells (Fig. 4F, Fig. S4A) and p53-mutated H23 cells (Fig. 4G). According to earlier studies showing that p53 is activated by glucose insufficiency [25, 37], we further focused whether p53 is involved in the regulation of PRMT1 in response to conditions of glucose deficiency. Indeed, PRMT1 level was inhibited by wild type p53, not mutp53(R175H, R248Q and R273H) in NSCLC cells (Fig. S4B). Meanwhile, PRMT1 is not regulated by glucose in the context of p53 mutation (Fig. S4C, D). Next, increased PRMT1 protein was observed following treatment with the p53 inhibitor pifithrin-α in A549 cells with glucose insufficiency (Fig. 4H). Moreover, p53 regulated PRMT1 protein but not mRNA expression (Fig. S4E, F). A549 cells with p53 overexpression were further treated with CHX or MG132. The results indicated that p53 mediates the degradation of PRMT1 protein (Fig. 4I, J). Furthermore, PRMT1 ubiquitination was increased with p53 overexpression (Fig. 4K). The ubiquitylation level of PRMT1 showed a significant decline upon treatment with pifithrin-α under conditions of glucose insufficiency (Fig. 4L). Based on the collective findings, we proposed that p53 mediated the degradation of PRMT1 protein in response to glucose insufficiency.

### Glucose inhibits ubiquitylation of PRMT1 through promoting USP7 expression in p53 wild-type NSCLC cells

**Fig. 5:**
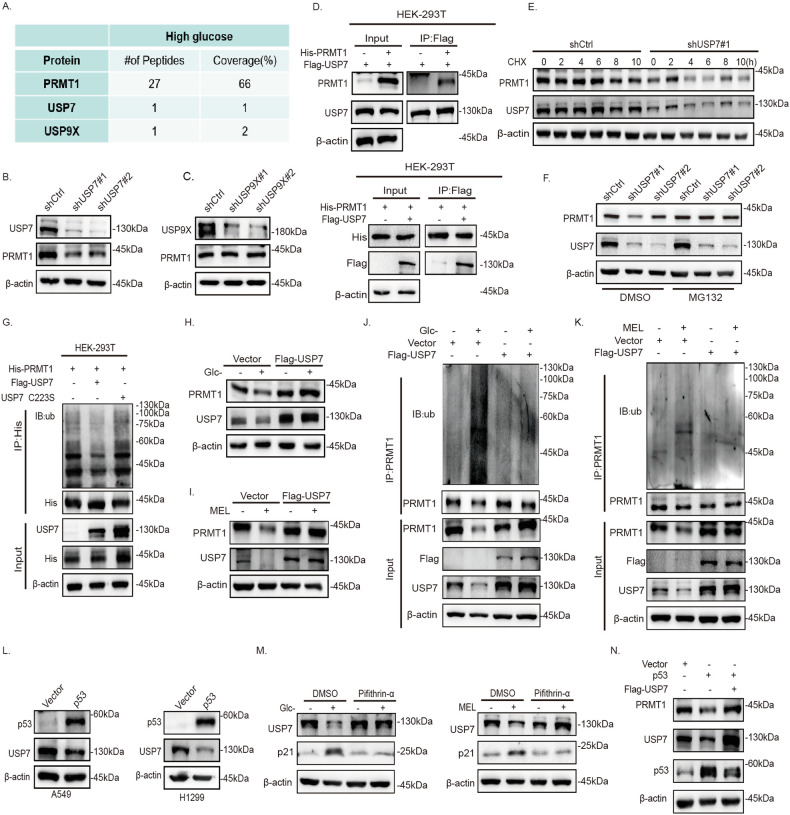
Glucose inhibits ubiquitylation of PRMT1 through promoting USP7 expression in p53 wild-type NSCLC. **A** Mass spectrometry analysis of Flag-PRMT1 protein complex purified from A549-Flag-PRMT1 cells via anti-Flag immunoaffinity chromatography. **B** Western blot analysis of PRMT1 in A549-shCtrl, shUSP7#1, and shUSP7#2 cells. **C** Western blot analysis of PRMT1 in A549-shCtrl, shUSP9X#1, and shUSP9X#2 cells. **D** Interactions between PRMT1 and USP7 detected via co-immunoprecipitation (Co-IP) assay with anti-Flag or anti-His antibodies in HEK-293T cells transfected with the Flag-USP7 and His-PRMT1 constructs, respectively. **E**, **F** Western blot analysis of PRMT1 in A549-shCtrl, A549-shUSP7#1, and A549-shUSP7#2 cells treated with CHX (100 μg/mL) or MG132 (10 μM). **G** HEK-293T cells were transfected with USP7 WT or USP7 C223S and cell lysates immunoprecipitated using anti-His antibody, followed by immunoblotting analysis. **H**, **I** Western blot analysis of PRMT1 in A549-Flag-USP7 cells subjected to glucose deprivation and treated with melatonin. **J**, **K** Western blot analysis of PRMT1 ubiquitination after immunoprecipitation with PRMT1 antibody in A549-Vector and A549-Flag-USP7 cells subjected to glucose deprivation or melatonin treatment. **L** Western blot analysis of USP7 in A549 and H1299 cells with p53 overexpressed. **M** Expression of USP7 in A549 cells treated with pifithrin-α under glucose insufficiency. **N** Western blot analysis of PRMT1 protein expression in A549-Vector, A549-p53, and A549-p53+Flag-USP7 cells.

To further clarify the mechanism underlying ubiquitination-mediated degradation of PRMT1 under glucose insufficiency, we performed mass spectrometry (MS) analysis of a 3×Flag-PRMT1 complex (Fig. S5A). PRMT1-interacting proteins (Table S3), USP7 and USP9X, were identified via MS in A549 cells (Fig. 5A). Among the two interacting proteins, knockdown of USP7 in A549 cells decreased the protein level of PRMT1 (Fig. 5B, C). PRMT1-USP7 interactions were further confirmed via interactive co-immunoprecipitation (Fig. 5D, Fig. S5B). To further determine the ability of USP7 to inhibit PRMT1 ubiquitination-mediated degradation, we treated A549-shUSP7 cells with MG132 or CHX. Our results indicated that USP7 promotes the stability of PRMT1 protein (Fig. 5E, F). Moreover, overexpression of USP7 WT, but not USP7 C223S, significantly weakened PRMT1 ubiquitination in HEK-293T cells (Fig. 5G). Since the level of USP7 was correlated with the concentration of glucose (Fig. S5C), USP7 expression was rescued in glucose-insufficient A549 cells, which ultimately led to an increase in PRMT1 expression (Fig. 5H, I) and a decrease in the ubiquitylation level of PRMT1 (Fig. 5J, K). The collective results indicate that USP7 deubiquitinates and stabilizes PRMT1 under high glucose conditions.

Based on the finding that p53 overexpression induced downregulation of USP7 in A549 and H1299 cells (Fig. 5L) and USP7 expression was significantly increased upon the addition of pifithrin-α when cells were grown under glucose insufficiency (Fig. 5M), we further investigated the involvement of USP7 in modulation of PRMT1 by p53. Overexpression of USP7 completely suppressed the expression of p53 and its inhibitory effect on PRMT1 in A549 cells (Fig. 5N). Taken together, our findings suggest that glucose insufficiency inhibits USP7 expression through activation of p53, further promoting ubiquitination-mediated degradation of PRMT1 in p53 wild-type NSCLC cells.

### Combined treatment with USP7 inhibitor and melatonin induces additive anti-NSCLC activity

**Fig. 6:**
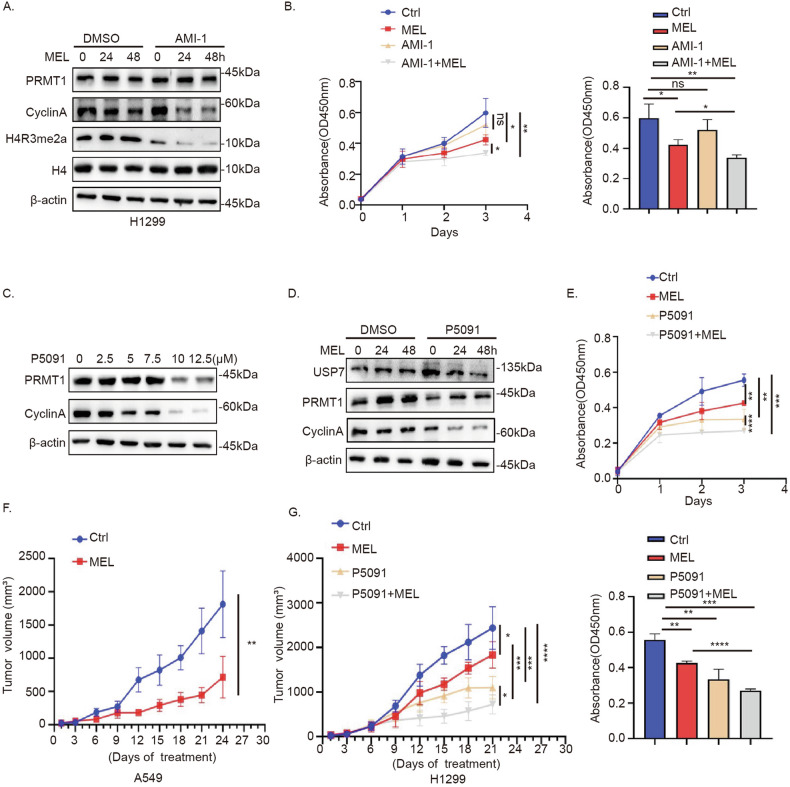
Combined treatment with a USP7 inhibitor and melatonin induces additive anti-NSCLC activity. **A** Western blot analysis of Cyclin A in AMI-1 (50 μM) and melatonin (2 mM)-treated H1299 cells. **B** Proliferation rates of H1299 cells treated with AMI-1 and melatonin evaluated via the CCK-8 assay. **C** H1299 cells were treated with the indicated amounts of P5091 for 48 h and PRMT1 protein levels determined via immunoblotting. **D** Western blot analysis of Cyclin A in H1299 cells treated with P5091 (10 μM) and melatonin. **E** Proliferation rates of P5091- and melatonin-treated H1299 cells evaluated via the CCK-8 assay. **F** Determination of tumor growth over time in nude mice (5 mice/group) transplanted with A549 cells and treated with melatonin (20 mg/kg). **G** Determination of tumor growth over time in nude mice (5 mice/group) transplanted with H1299 cells and treated with melatonin (20 mg/kg), P5091 (10 mg/kg), or a combination of the two drugs. Error bars, mean ± SD. ns (non-significant), *P* > 0.05; *P < 0.05; **P < 0.01; ***P < 0.001; ****P < 0.0001.

Based on the finding that melatonin significantly inhibited cell proliferation through inhibiting PRMT1 expression in p53-wild NSCLC cells (Fig. 1, Fig. S6A, C) but had no effect on PRMT1 protein in p53-deficient and p53-mutated NSCLC (Fig. 4), we hypothesized that inhibition of PRMT1 could further enhance the anticancer effects of melatonin. To examine this hypothesis, p53-deficient H1299 cells were treated with a combination of AMI-1 and melatonin. Interestingly, Cyclin A2 was significantly downregulated following co-treatment (Fig. 6A). Moreover, proliferative ability was markedly reduced in H1299 cells, as evident from CCK-8 viability (Fig. 6B) and colony formation assays (Fig. S6C). Our findings confirm that suppression of PRMT1 effectively enhances the inhibitory effect of melatonin on proliferation of p53-deficient NSCLC cells.

USP7 is a known druggable therapeutic target. Our initial results showed a dose-dependent reduction in PRMT1 protein levels following treatment with the USP7 inhibitor P5091 in H1299 cells (Fig. 6C). Co-treatment with P5091 and melatonin significantly suppressed H1299 cell proliferation (Fig. 6D, E). To establish the therapeutic efficacy of melatonin and P5091, A549 and H1299 cells were implanted subcutaneously in nude mice, and treated with melatonin, P5091 or melatonin in combination with P5091. Notably, melatonin induced a marked reduction in proliferation of p53 wild-type NSCLC cells (Fig. 6F) while the combination of P5091 and melatonin elicited strong growth inhibition of p53-deficient NSCLC cells (Fig. 6G). These results indicate that the combined actions of the USP7 inhibitor and melatonin exert an additive therapeutic effect against p53-deficient NSCLC.

In addition, the gene expression level of PRMT1 and USP7 in several tumor types was analyzed by TIMER2.0 network tool. The results showed that the gene expression of PRMT1 and USP7 in NSCLC was higher than that in normal tissues (Fig. S9A). And the gene expression of PRMT1 and USP7 in p53 mutated NSCLC was higher than that in p53 non-mutated tissues (Fig. S9B). High PRMT1 and USP7 expression predicted a poor prognosis (Fig. S9C). These results indicated that high PRMT1 and USP7 expression in NSCLC is likely correlated to more aggressive tumor characteristics.

## Discussion

In this study, we have described a novel mechanism whereby p53 acts as a sensor of glucose to regulate the Warburg effect. Specifically, p53 mediates PRMT1 protein downregulation, leading to inhibition of the Warburg effect and, in turn, suppression of NSCLC cell proliferation under conditions of glucose insufficiency. PRMT1 promotes the gene transcription of PTBP1 and inhibits expression of PKM1, which ultimately induces glycolysis. To our knowledge, this is the first report to identify a critical role of PRMT1 on the Warburg effect in NSCLC cells as well as clarify the series of molecular events involved in this process.

Although PRMT1 is implicated in cellular metabolism, its function in Warburg effect has not been established. Our results demonstrated that PRMT1 increases the PKM2/PKM1 mRNA ratio through upregulation of PTBP1, in turn, promoting the Warburg effect in NSCLC cells. However, in addition to PTBP1, hnRNPA1 and hnRNPA2B1 are involved in regulating the alternative splicing of PKM as RNA-binding proteins [35, 36]. Screening of mass spectrometry binding data showed that hnRNPA1 and hnRNPA2B1 interact with PRMT1 in NSCLC cells. Asymmetric dimethylation of hnRNPA1 by PRMT1 is reported to suppress endogenous hnRNPA1 ITAF activity in HeLa cells [38]. Arginine-methylated hnRNPA1 by PRMT4/5/7 is involved in hnRNPA1 binding with pre-mRNAs and alternative splicing events [39]. Further research is required to determine whether methylated hnRNPA1/hnRNPA2B1 is involved in PKM pre-mRNA splicing in a synergistic manner with PTBP1 that is upregulated by PRMT1.

p53 is activated in response to external environmental factors, such as nutrient starvation. On activation, p53 mediates important cellular processes, such as cell death, cell cycle arrest and metabolic reprogramming [40–42]. Data from the current study showed that activation of p53 caused by insufficient glucose supply promotes the ubiquitination-mediated degradation of PRMT1 in NSCLC cells. Under insufficient glucose availability, the level of USP7 is inhibited by p53, resulting in degradation of PRMT1 via ubiquitination of the proteasome pathway, thereby inhibiting the Warburg effect and proliferation of NSCLC cells. However, the specific mechanism of USP7 regulation by p53 requires further exploration. We have identified PRMT1 as a novel target for p53 for the first time, providing new insights into the mechanism underlying p53-mediated regulation of the Warburg effect in NSCLC. Interestingly, earlier reports have demonstrated that knockdown of PRMT1 in cardiac epicardial cells regulates Mdm4 splicing and promotes p53 protein stability [28]. In addition, results from the current study showed that PRMT1 inhibits protein stabilization of p53 in NSCLC (Supplementary Fig. S8A, B). These findings suggest a reciprocal regulation between PRMT1 and p53.

Melatonin increases p53 activity for driving cells destined for apoptosis/growth inhibition in cancer cells [43]. According to the Cancer Genome Atlas (TCGA) database on the cBio Cancer Genome website, loss of p53 function is widely detected in approximately 68% of NSCLC cases [44]. In this study, PRMT1 and USP7 levels were not affected by melatonin in p53-deficient H1299 cells. Combined usage of melatonin and USP7 inhibitors was superior to melatonin alone in the treatment of p53-deficient NSCLC. However, p53 mutations are commonly present in more than 50% of all cancers. Mutation of p53 not only leads to loss of anti-tumor transcriptional activity but also often gain of cancer-promoting function, ultimately enhancing tumor proliferation, invasion and drug resistance. Whether PRMT1-based treatment can improve the prognosis of p53-mutated NSCLC needs further exploration.

**Fig. 7:**
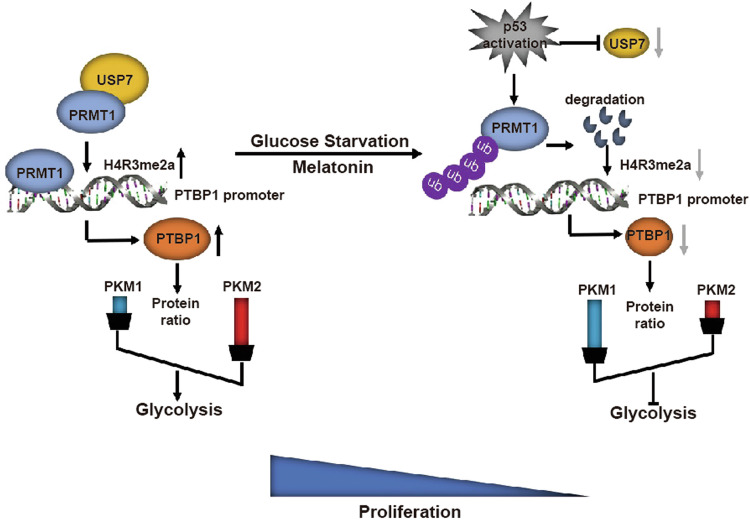
A working model. Upon glucose intake, USP7 binds to PRMT1 protein to facilitate maintenance of its stability. PRMT1 increases the PKM2/PKM1 ratio through promoting gene transcription of PTBP1, which augments aerobic glycolysis and cell proliferation in NSCLC. Conversely, under glucose insufficiency, p53 is activated to inhibit USP7 expression, further promoting PRMT1 protein degradation and inhibiting aerobic glycolysis and proliferation in p53 wild-type NSCLC cells.

Conclusively, data acquired from this study support the working model involving a series of molecular events depicted Fig. 7. The PRMT1 protein is stabilized through USP7-mediated deubiquitylation upon glucose intake. PRMT1 promotes the Warburg effect by upregulating PTBP1 to increase the ratio of PKM2/PKM1, which subsequently promotes NSCLC cell proliferation. However, activation of p53 mediates protein degradation of PRMT1 through inhibiting the level of USP7 under glucose insufficiency. For p53-deficient NSCLC, combination treatment with melatonin and the USP7 inhibitor P5091 could significantly inhibit tumor growth. Based on the collective findings, we propose a novel mechanism by which p53 regulates a metabolic shift in NSCLC cells facing insufficient energy supply and a promising therapeutic strategy for p53-deficient NSCLC.

## Materials and methods

### Cell cultures

Human cell lines (A549, H1299, H23, HEK-293T) were used in the present study. HEK-293T, A549 and H1299 cell lines purchased from the ATCC (Manassas, VA, USA) were cultured in high-glucose Dulbecco’s modified Eagle’s medium (Gibco) containing 10% FBS. H23 cells were gifted by the Institute of Nutrition and Health (CAS). H23 cells were cultured in RPMI1640 (Gibco) containing 10% FBS. All cell lines were grown at 37 °C with 5% CO_2_.

### Reagents, antibodies, and plasmids

A description of reagents, antibodies and plasmids is provided in Supplementary Materials and Methods and Supplementary table 1.

### Immunoprecipitation and western blot analyses

Cells were harvested and lysed in buffer A (25 mM Tris-HCl pH 8.0, 10 mM NaCl, 0.5 mM EDTA, 0.5% NP-40) containing a protease inhibitor cocktail tablet (Roche) and shaken on ice for 35 min with an interval of 3 min. Total protein lysates were incubated overnight with 3.5 μg antibodies with gentle shaking at 4 °C, followed by 30–40 μL Pure Proteome Protein A/G Mix Magnetic Beads (Millipore, Cat.No.LSKMAGAG02) for 2–4 h. Beads were washed with buffer A, resuspended in 40–50 μL 2× loading buffer, and incubated for 8–10 min at 100 °C. After disposal of the beads, protein lysates were subjected to SDS–PAGE, transferred to hydrophobic PVDF membrane, and visualized with ECL reagent.

### RNA extraction, reverse transcription, and real-time RT-PCR

Detailed protocols have been reported previously by our group [45]. The experimental methods are described in Supplementary Materials and Methods. And the gene-specific primer sequences for PCR were provided in Supplementary Table 2.

### Seahorse assays

Extracellular acidification rate measurement (ECAR) and oxygen consumption rate measurement (OCR) were measured using an XFp Extracellular Flux Analyzer (Seahorse Bioscience). Details of the methods are provided in the Supplementary Materials and Methods section.

### Lactate measurements

Two kits were employed to determine the lactic acid content: (1) Lactate Assay Kit (Sigma-Aldrich) and (2) L-lactic acid (L-LA) content detection kit (Solarbio). Lactate production was determined using a colorimetry/fluorescence-based assay kit following the manufacturer’s instructions.

### Pyruvate kinase assay

A549 and H1299 cells transfected with the sh-Ctrl, shPRMT1#1, and shPRMT1#2 constructs were seeded in 6-well plates and incubated for 24 h. PK activity in cell lysates was measured with a specific assay kit (Solarbio, BC0545) according to the manufacturer’s instructions.

### ATP measurement

A549 and H1299 cells were seeded in 6-well plates and incubated for 24 h. The ATP level was measured using an ATP Assay Kit (Beyotime, S0026) following the manufacturer’s instructions.

### RNA splicing assay

A549 and H1299 cells transfected with the sh-ctrl, shPRMT1#1 or shPRMT1#2 constructs were cultured in 10 cm plates. RNA was extracted and reverse-transcribed into cDNA using 30 cycles of PCR. The primers used for PCR were as follows: human PKM exon 8 forward, 5′-CTGAAGGCAGTGATGTGGCC-3′, and human PKM exon 11 reverse, 5′-ACCCGGAGGTCCACGTCCTC-3′. Amplified products (20 μL) were digested with *Pst*I (New England Biolabs, USA) for 1 h and the relative levels of PKM1 and PKM2 analyzed via 4% non-denaturing PAGE.

### Luciferase reporter assay

For the luciferase reporter assay, HEK293T cells were co-transfected with promoters of PTBP1 and Flag-PRMT1 for 48 h. Relative luciferase activities were measured using a Dual Luciferase Reporter Assay Kit (E1910; Promega). The following primer sequences were used:

PTBP1 full-length promoter (Sense: 5’-AAACGAGAATTGTCATGTCTTCTC-3’, Antisense: 5’-GGCTCCGAGTTATAGACTCACAAAA-3), PTBP1 truncated promoter plasmid primers (+100– −200 bp (P1): TTCGGCCTTGAGGAATAACCGCCT, +100– −500 bp (P2): TCAGTTTGAATCGGACTTTTTGGCC, +100–1000 bp (P3): AAGGTCCAGGCCTCAGTTTCCCCAG, +100–1600 bp (P4): CCTGGCCCTCAGTTTCCCC).

### Chromatin immunoprecipitation (ChIP) assay

Cells were collected and subjected to the formaldehyde cross-linking method reported by Hu et al. [46]. ChIP experiments were carried out in accordance with the manufacturer’s instructions (#9006; CST).

### Cell proliferation assay

A549 and H1299 cells were seeded in 96-well plates at a density of 500 cells/well. After 24 h, 10 μL CCK-8 solution (APEXBIO) was added to the medium and cells placed for 2–3 h in a 37 °C incubator. Proliferation was quantified by measuring absorbance at 450 nm. For the colony formation assay, 500 suitably treated A549 and H1299 cells were suspended in each well of a 6-well plate for culture. After two weeks, cells were stained with 0.1% crystal violet, imaged, and the number of colonies counted.

### Animal work

A549 (4 × 10^6^) or H1299 cells (4 × 10^6^) were subcutaneously injected into 5-week-old male BALB nude mice (*n* = 5 mice per group). Tumor volume (0.5 × length × width^2^) and body weight were measured over 24 days and data expressed as mean ± SD. For the single melatonin treatment, five mice per group were intraperitoneally administered either vehicle or 20 mg/kg melatonin every day for 4 weeks. Mice were monitored every 3 days and body weights and tumor growth assessed as described above. For combination treatment with melatonin and P5091, four groups of mice were evaluated. Five mice per group were intraperitoneally administered melatonin (20 mg/kg) every day for 4 weeks and P5091 (10 mg/kg) every day for 3 weeks. Mice were monitored every 3 days and body weights and tumor growth assessed as described above. All animal experiments were approved by the Ethical Committee of human and animal Experiments of Northeast Normal University, China (Authorization number: 202302019).

### Statistical analysis

Data are presented as means ± SD. Student’s t-test (two-tailed) was applied to determine the significance of differences between groups. Statistical analysis was conducted using GraphPad Prism 5 software.

### Supplementary information

Figure S1

Figure S2

Supplementary Materials and Methods

Supplementary Table 1

Supplementary Table 2

Supplementary Table 3

Figure S3

Figure S4

Figure S5

Figure S6

Figure S7

Figure S8

Figure S9

Full and uncropped western blots

## Data Availability

The data supporting the findings of this study are available from the corresponding author upon reasonable request.
