# Supplementary figures and images for: PRMT1 promotes Warburg effect by regulating the PKM2/PKM1 ratio in non-small cell lung cancer
Source: Cell Death Dis. 2024 Jul 15;15(7):504. doi: 10.1038/s41419-024-06898-x (PMC11251085; doi:10.1038/s41419-024-06898-x)

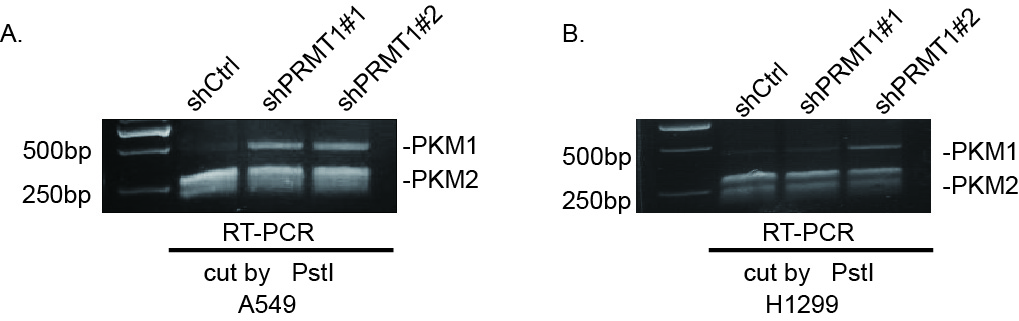

Supplement: Supplementary file 1 — Figure S1 [file 41419_2024_6898_MOESM1_ESM.jpg]

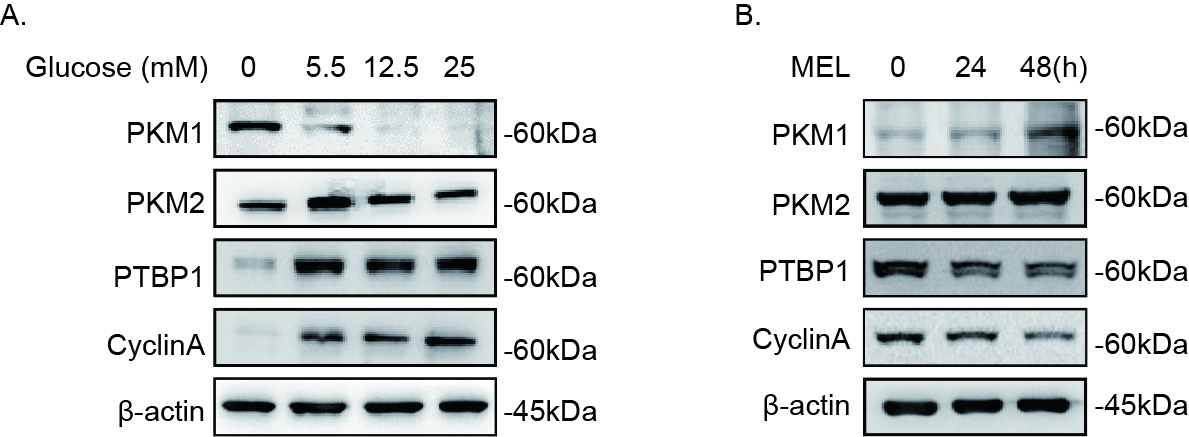

Supplement: Supplementary file 2 — Figure S2 [file 41419_2024_6898_MOESM2_ESM.jpg]

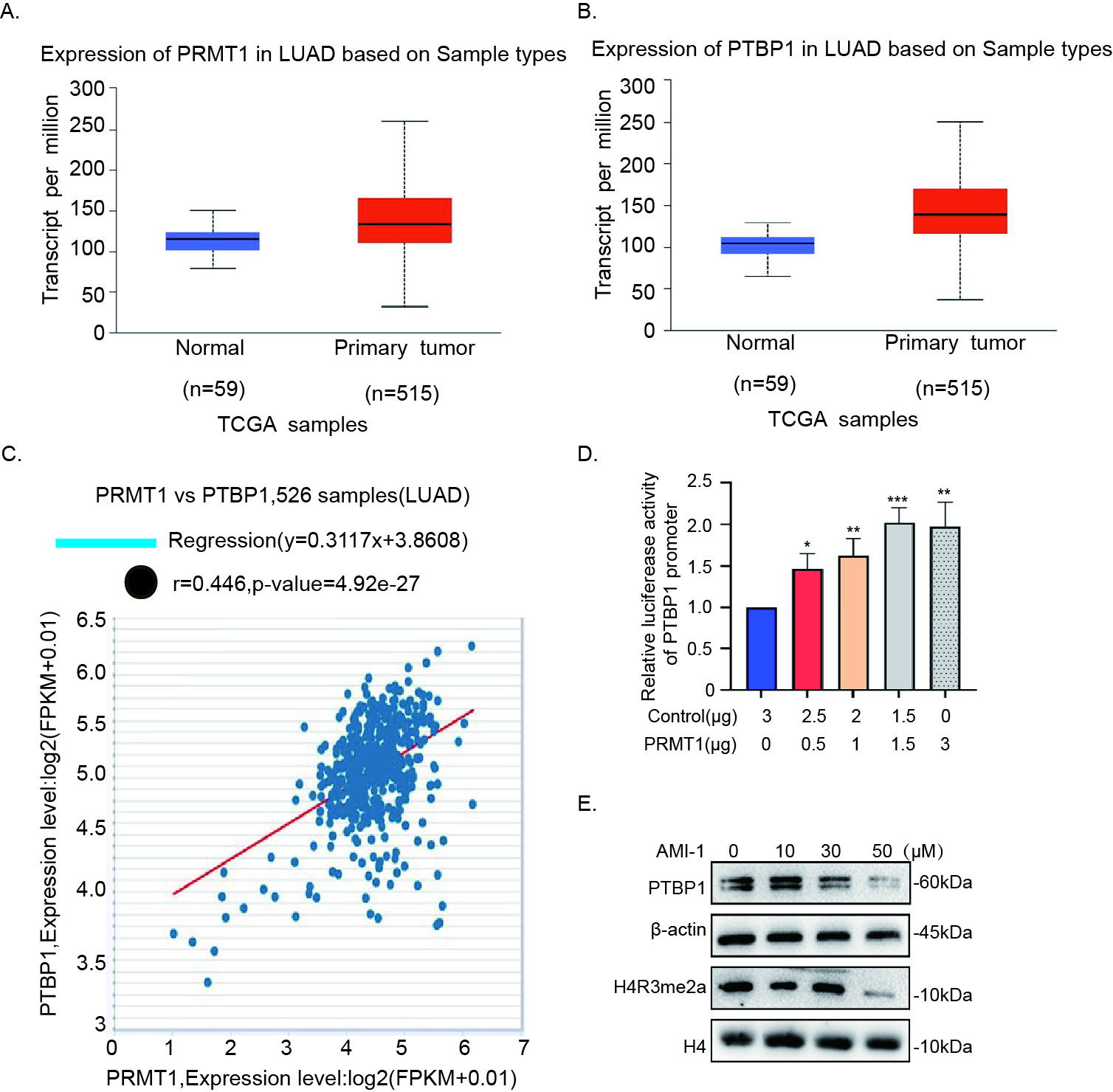

Supplement: Supplementary file 7 — Figure S3 [file 41419_2024_6898_MOESM7_ESM.jpg]

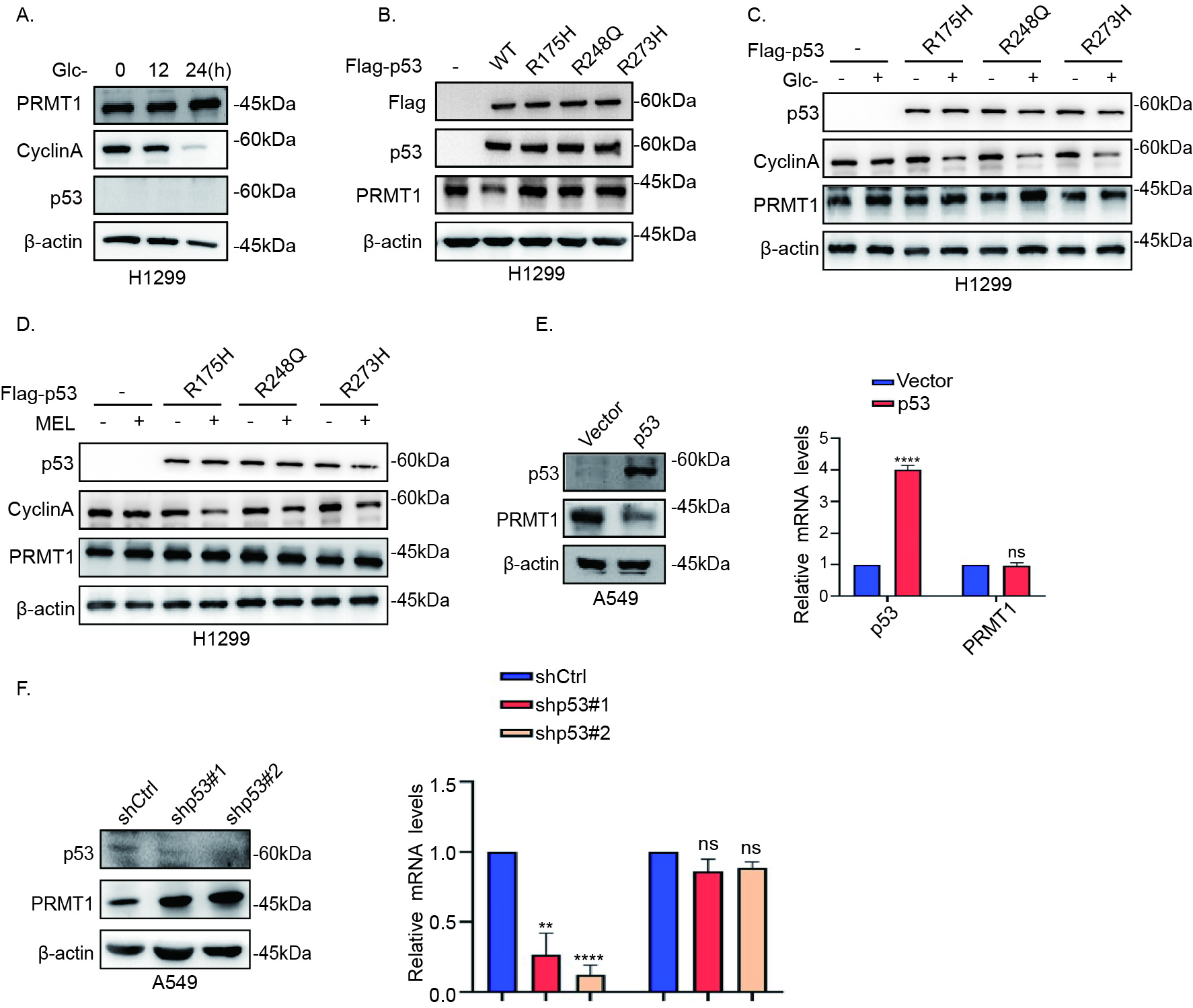

Supplement: Supplementary file 8 — Figure S4 [file 41419_2024_6898_MOESM8_ESM.jpg]

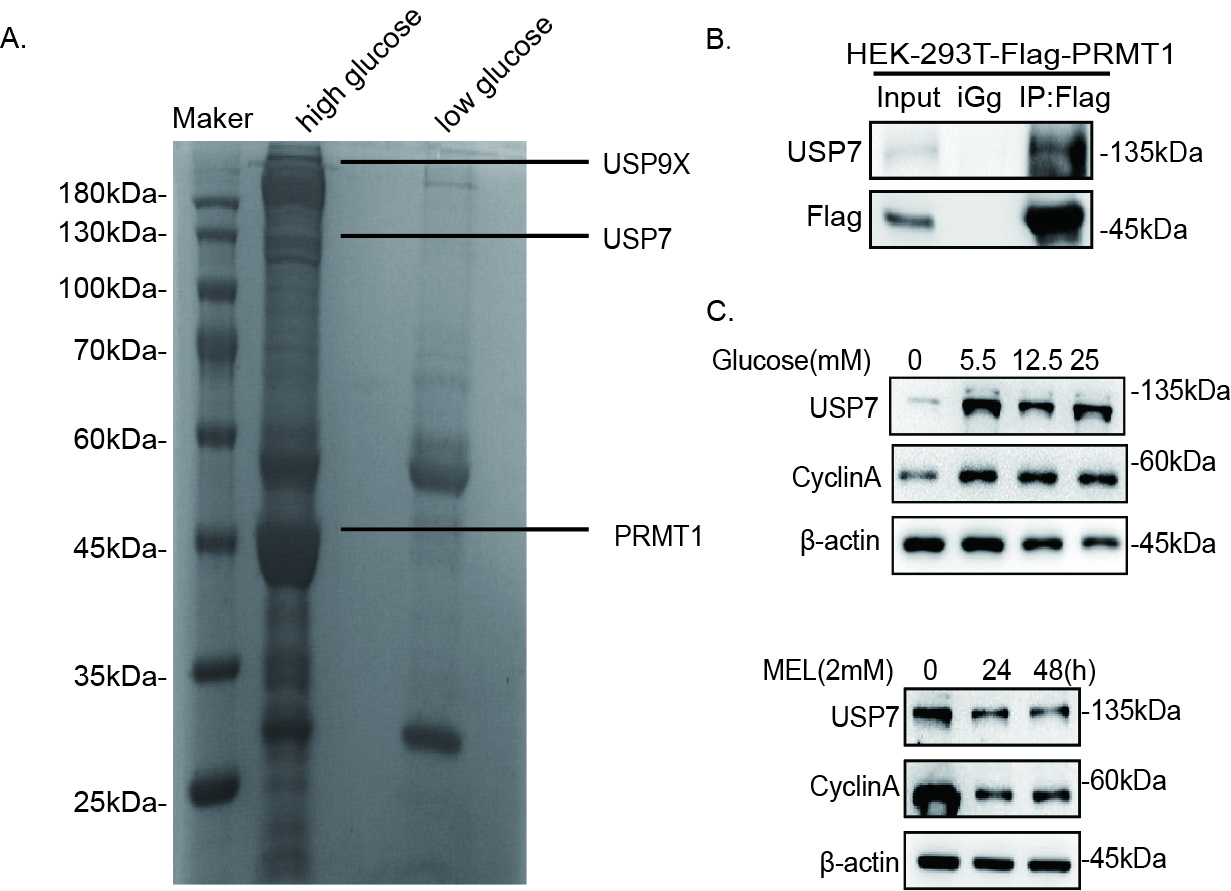

Supplement: Supplementary file 9 — Figure S5 [file 41419_2024_6898_MOESM9_ESM.jpg]

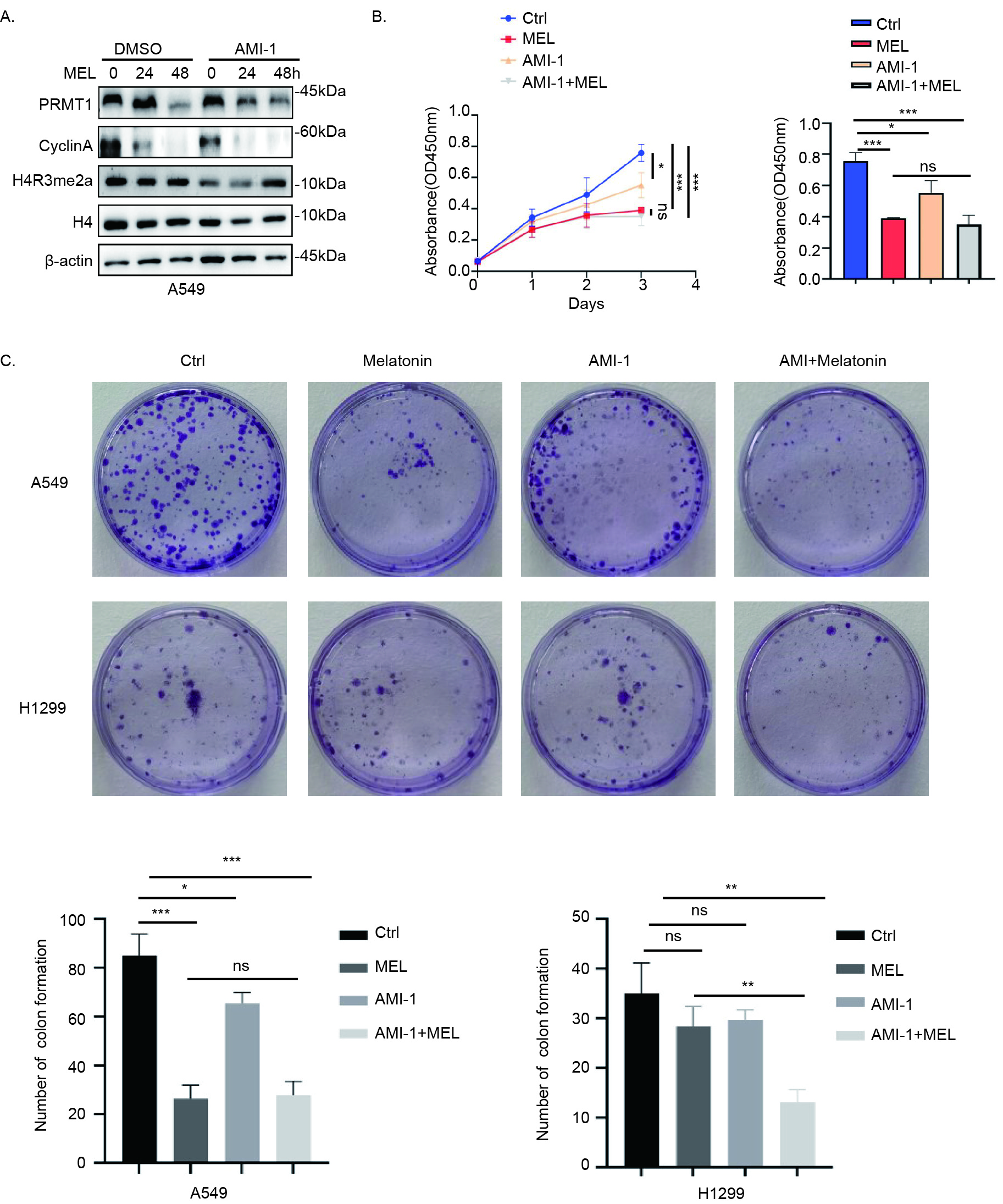

Supplement: Supplementary file 10 — Figure S6 [file 41419_2024_6898_MOESM10_ESM.jpg]

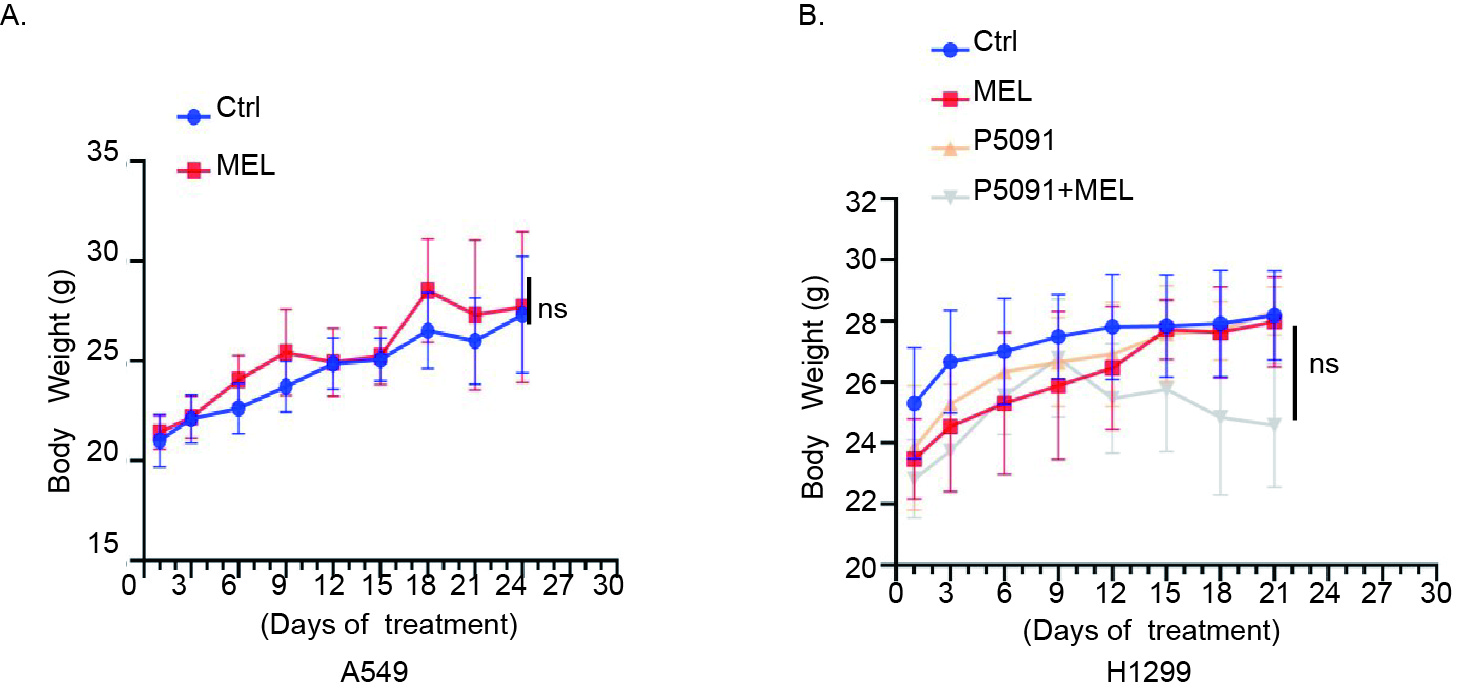

Supplement: Supplementary file 11 — Figure S7 [file 41419_2024_6898_MOESM11_ESM.jpg]

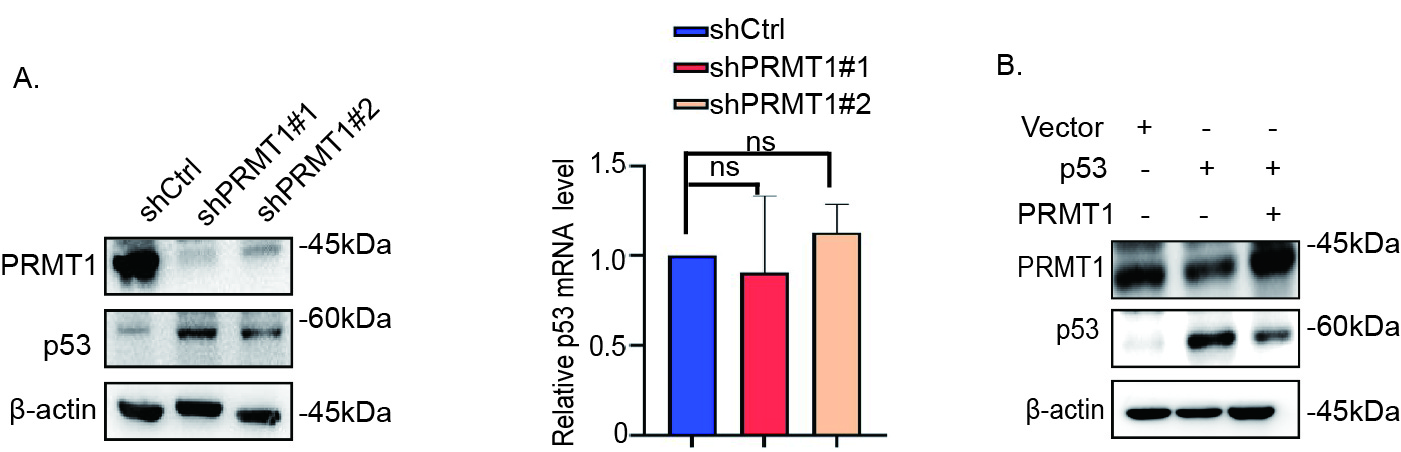

Supplement: Supplementary file 12 — Figure S8 [file 41419_2024_6898_MOESM12_ESM.jpg]

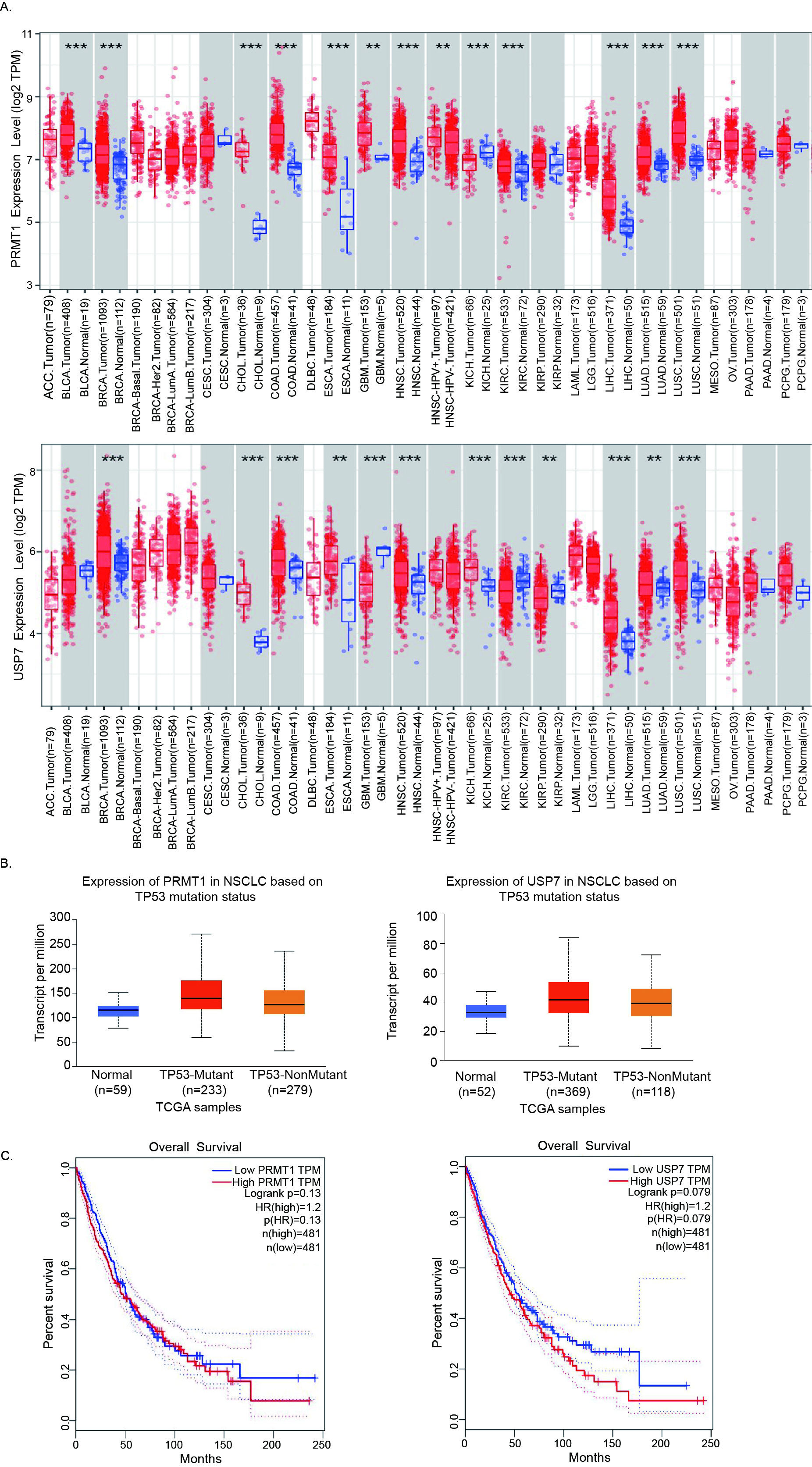

Supplement: Supplementary file 13 — Figure S9 [file 41419_2024_6898_MOESM13_ESM.jpg]

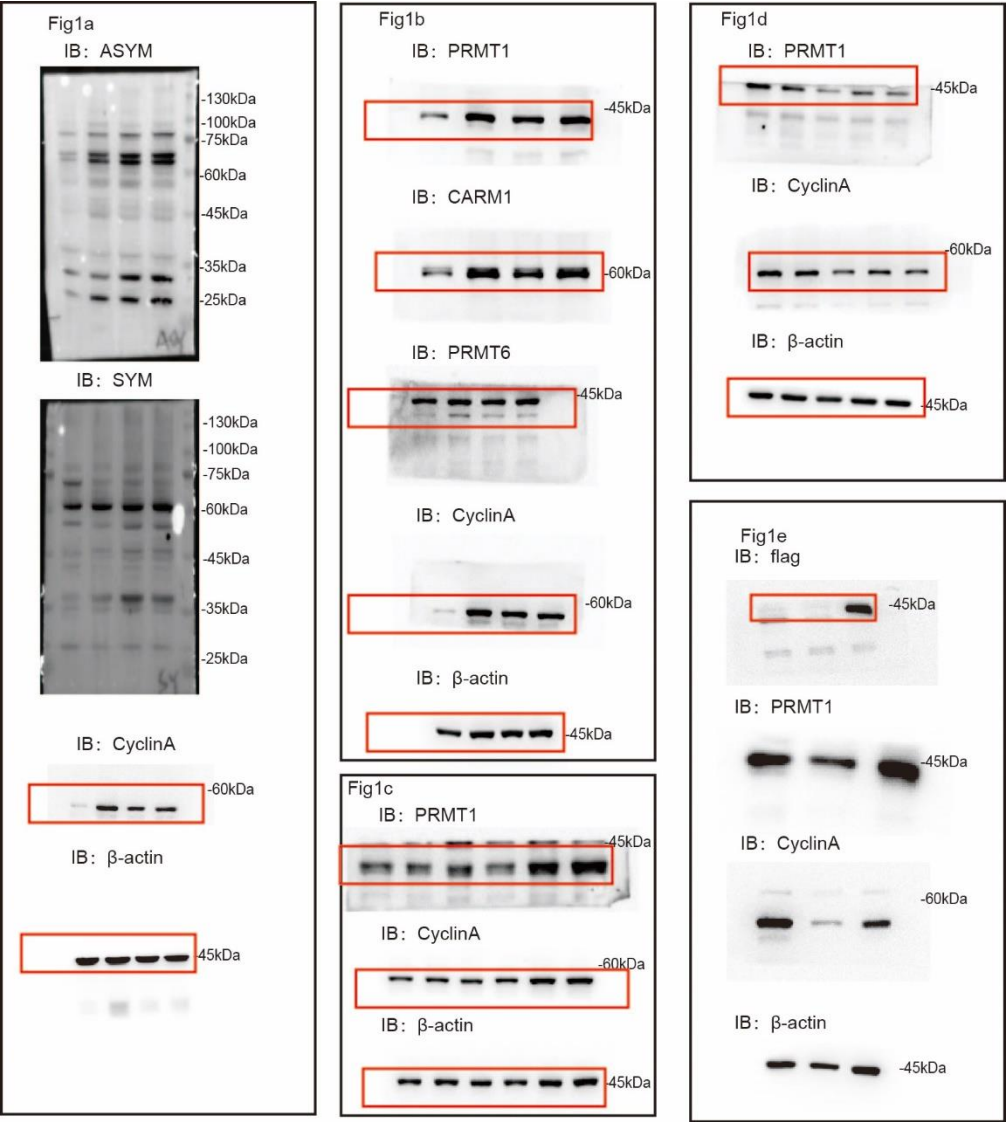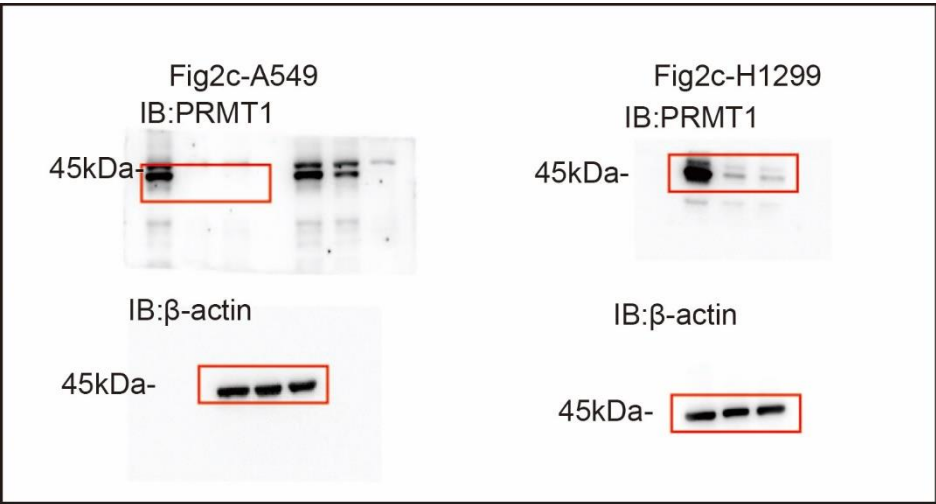

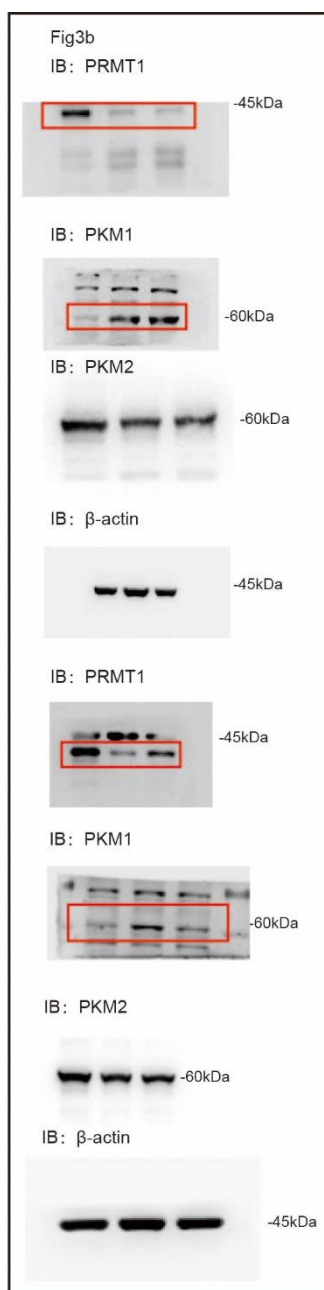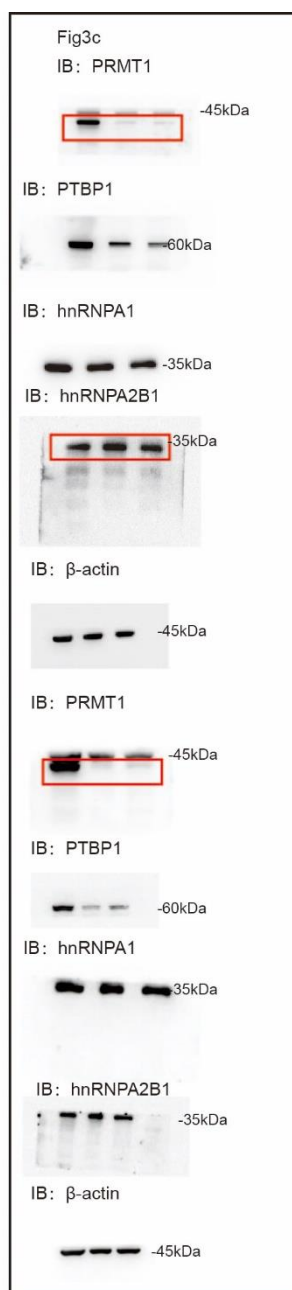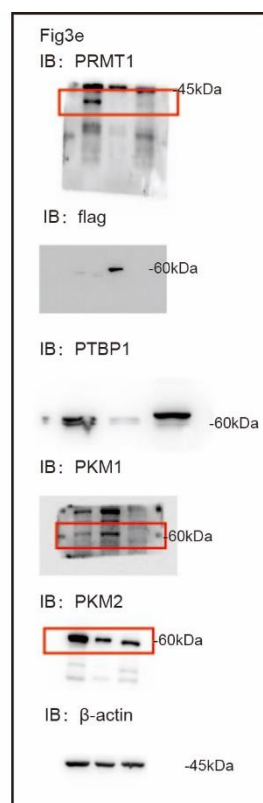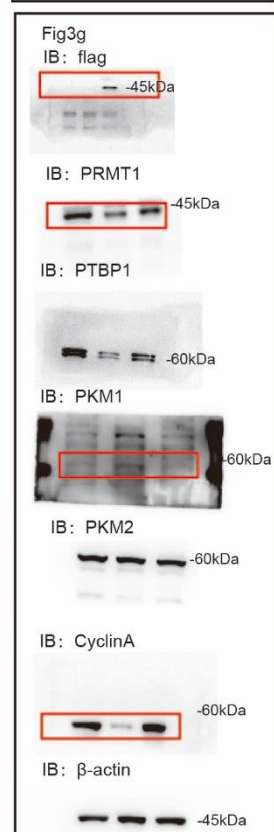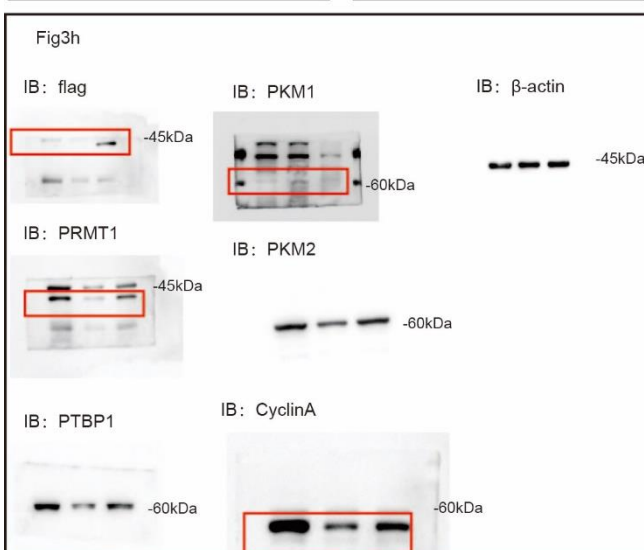

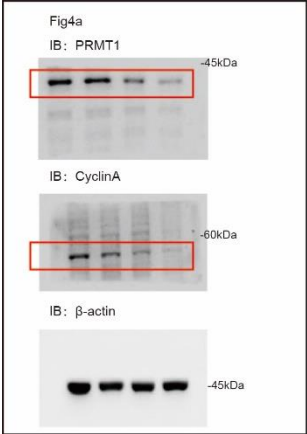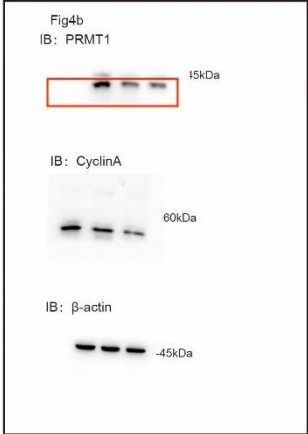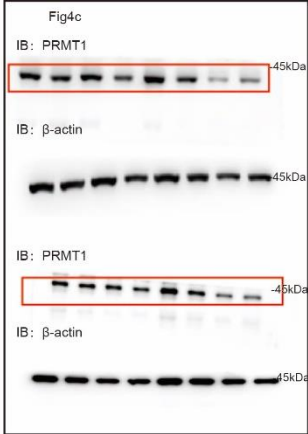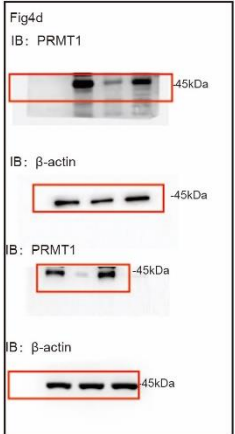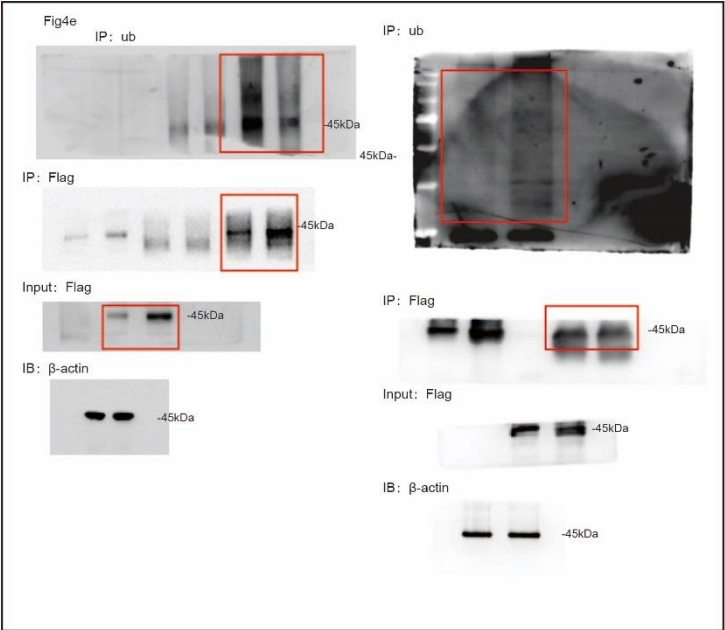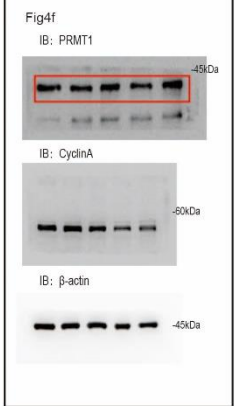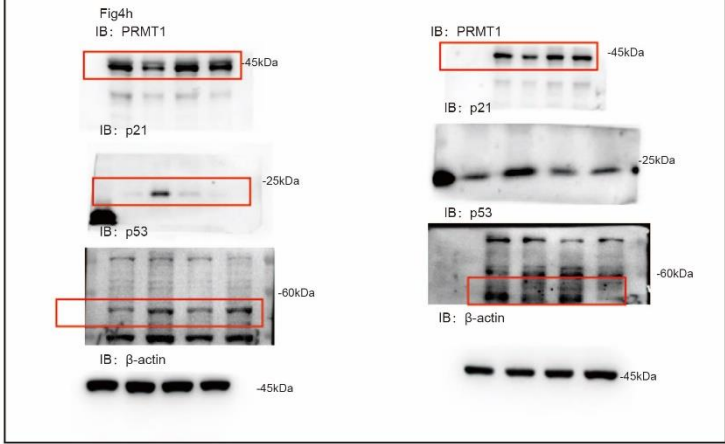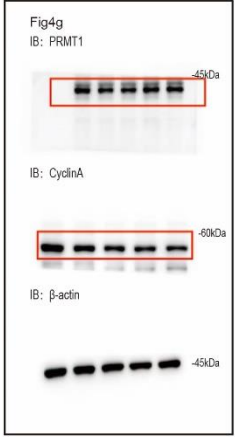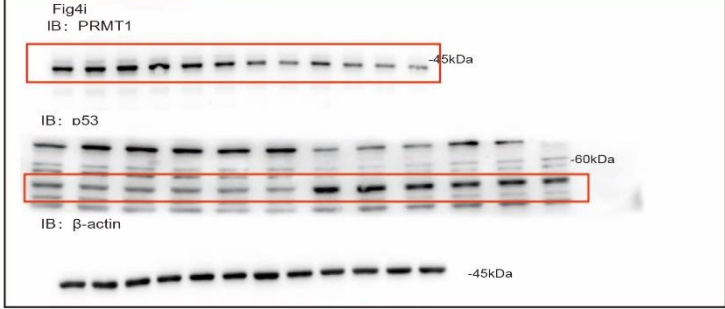

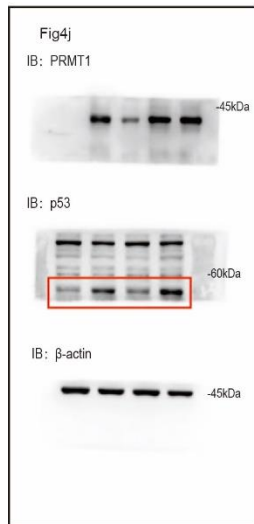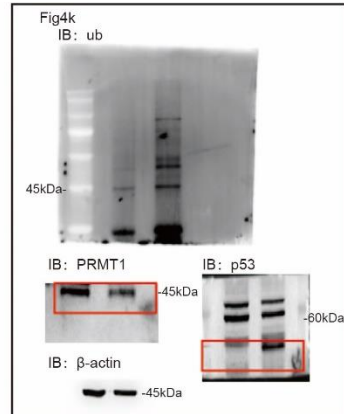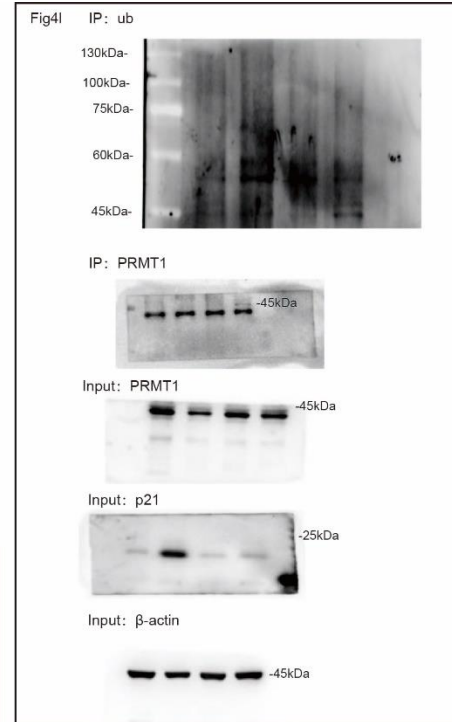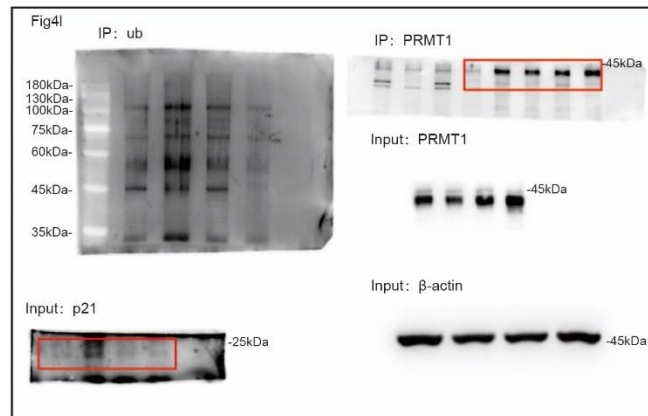

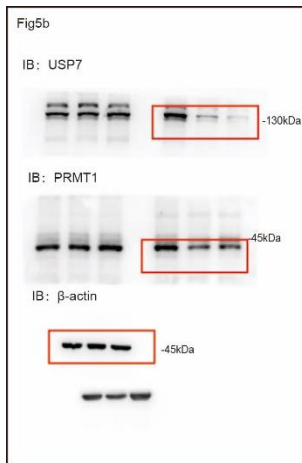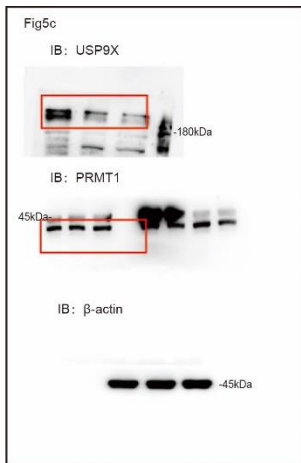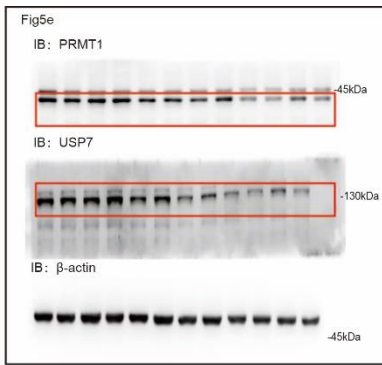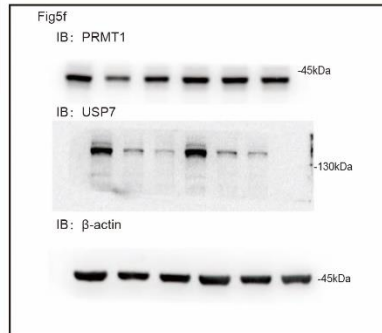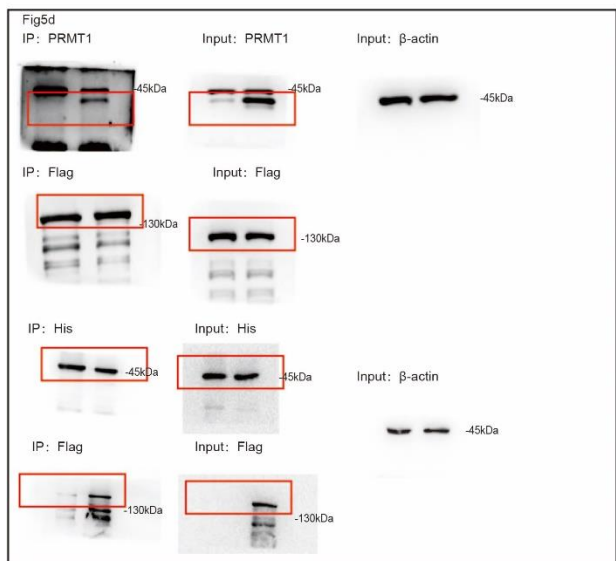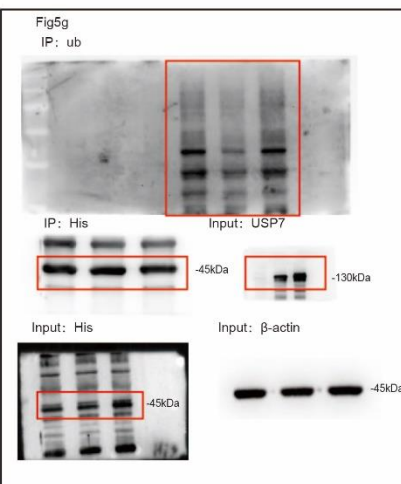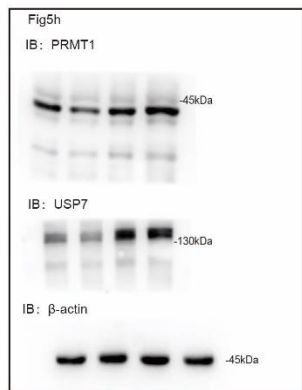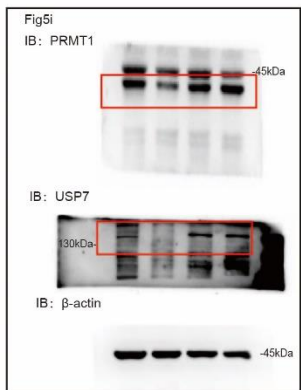

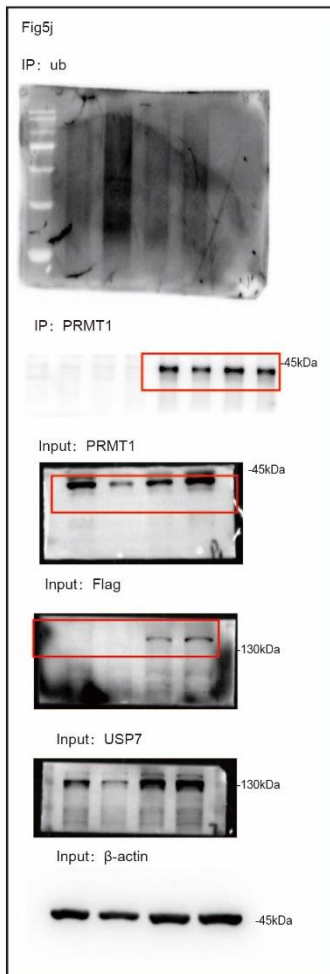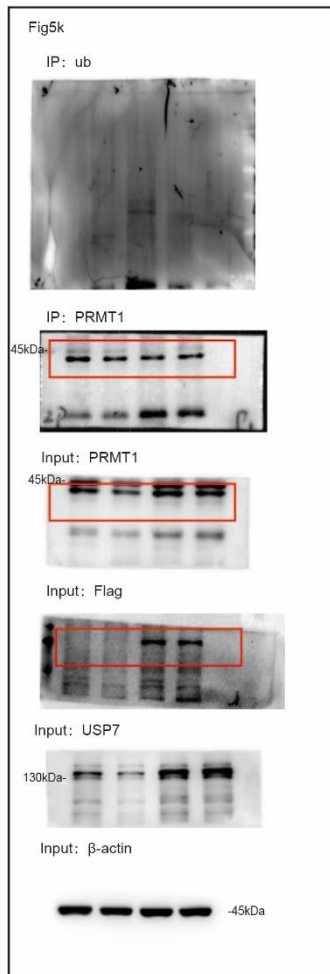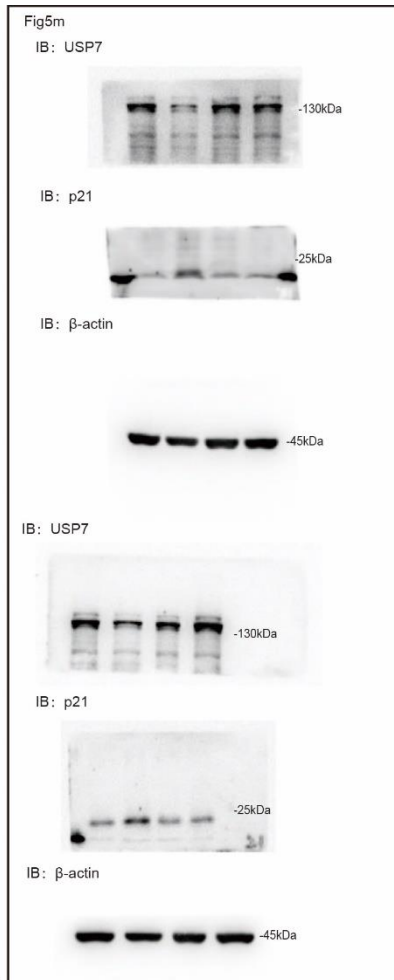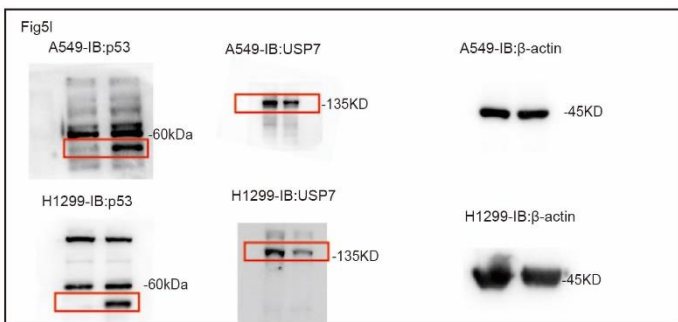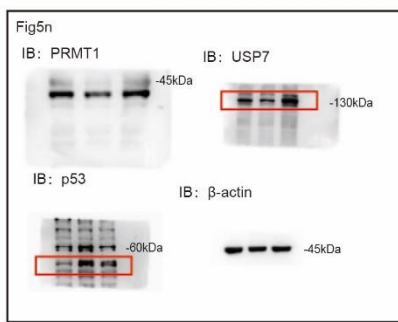

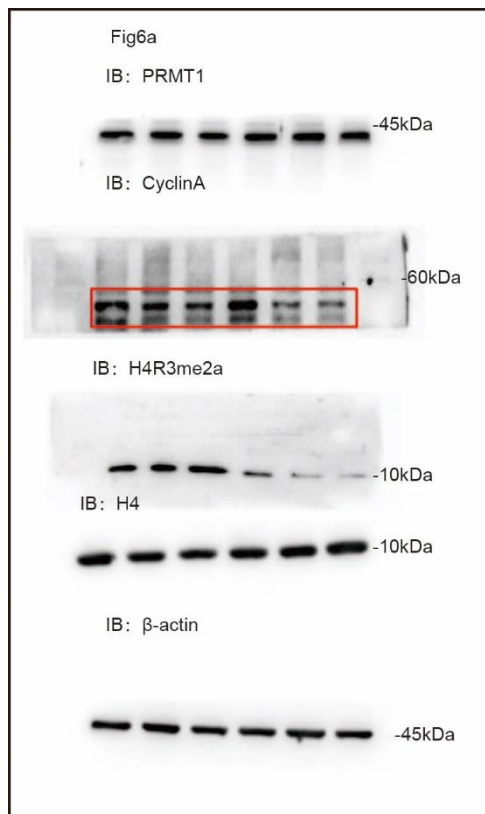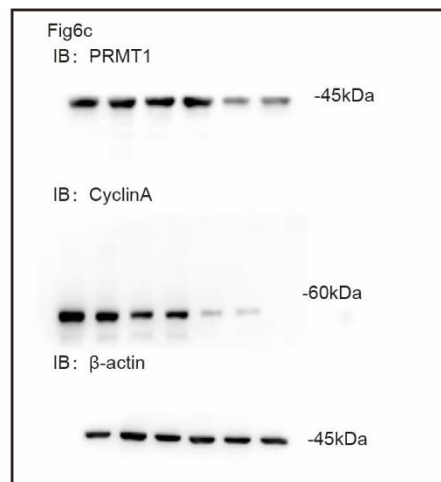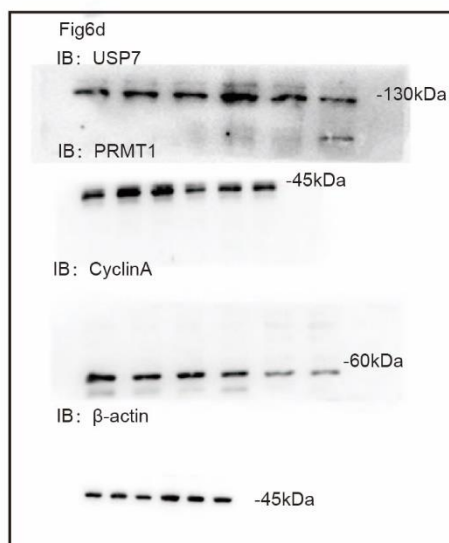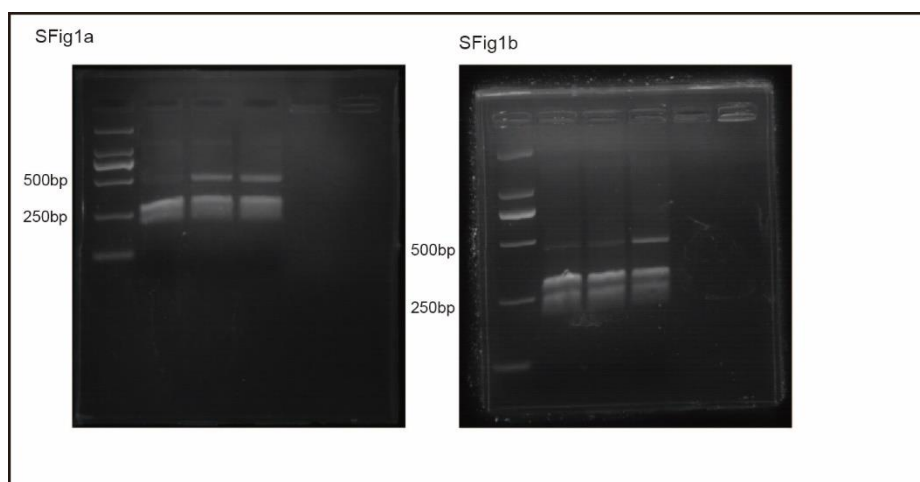

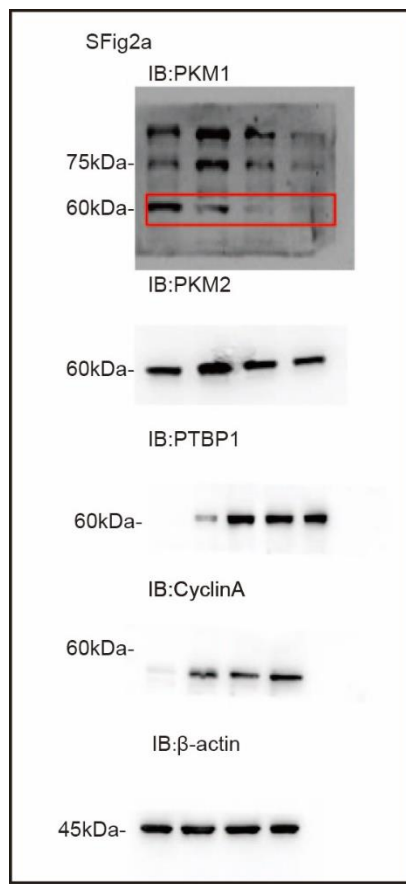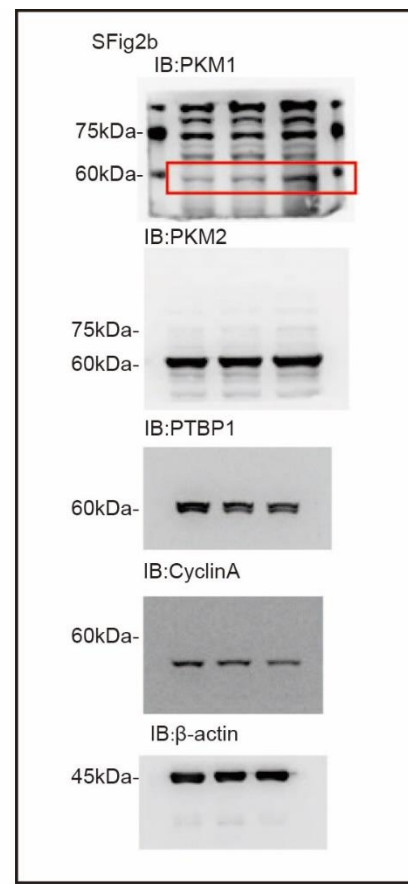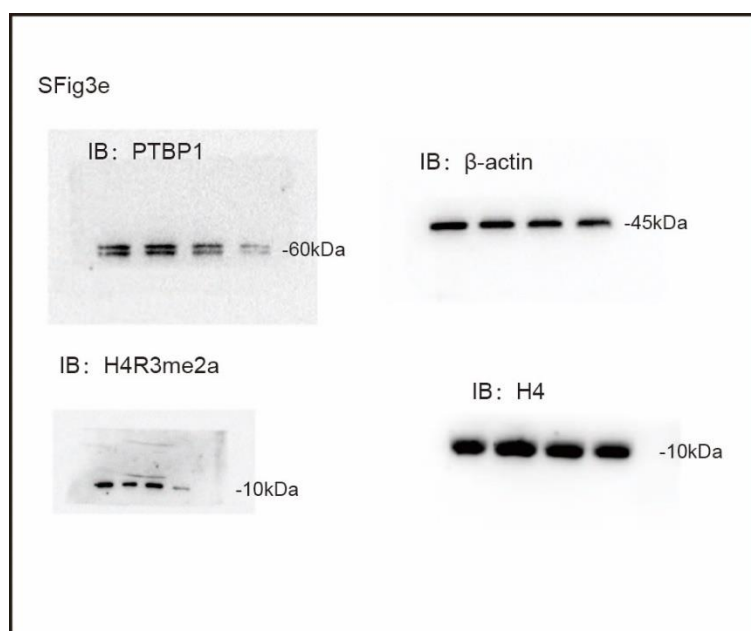

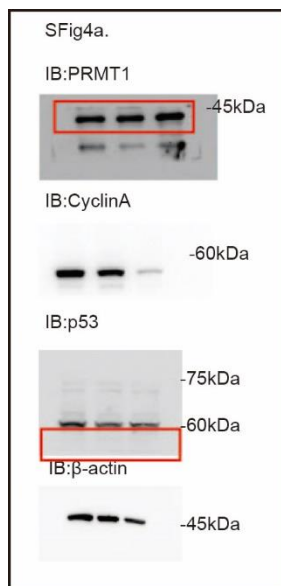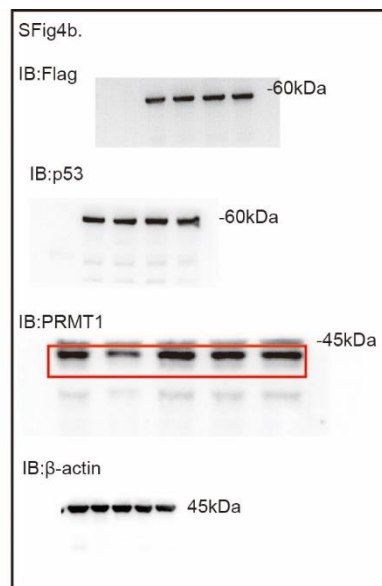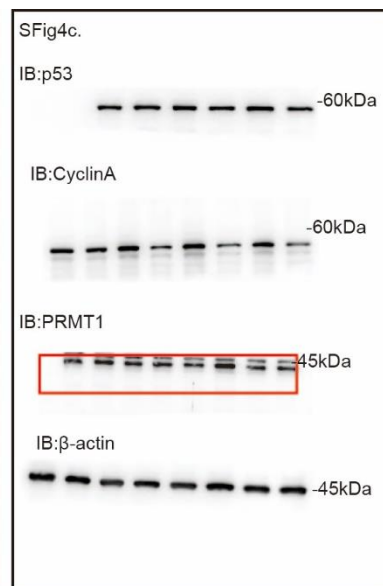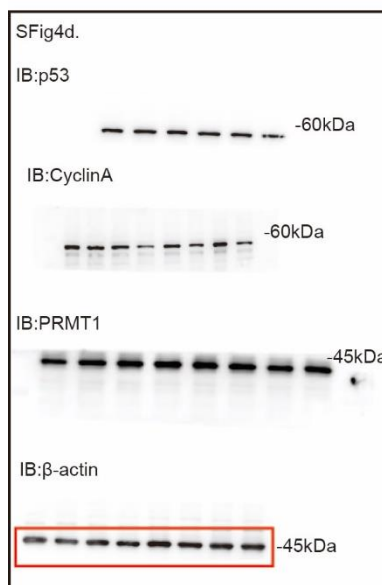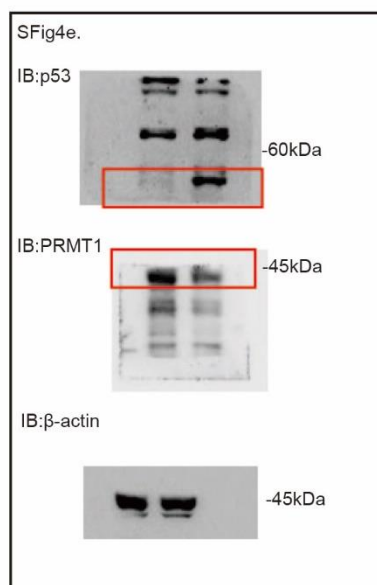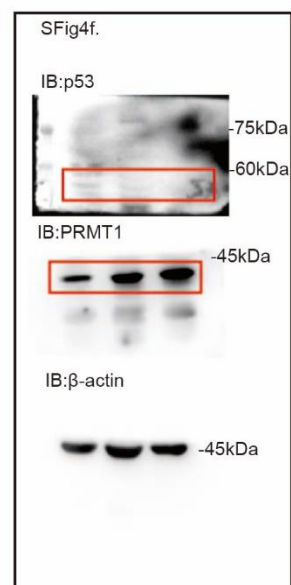

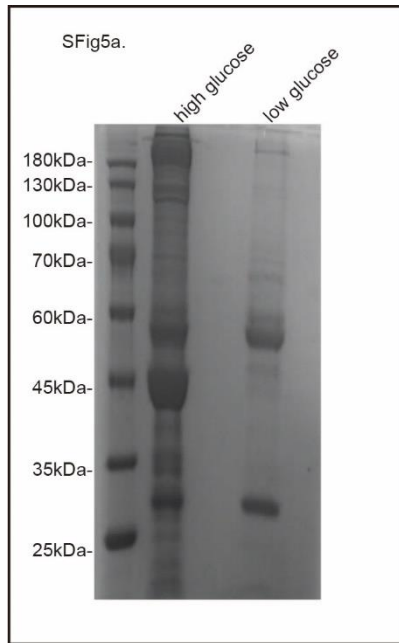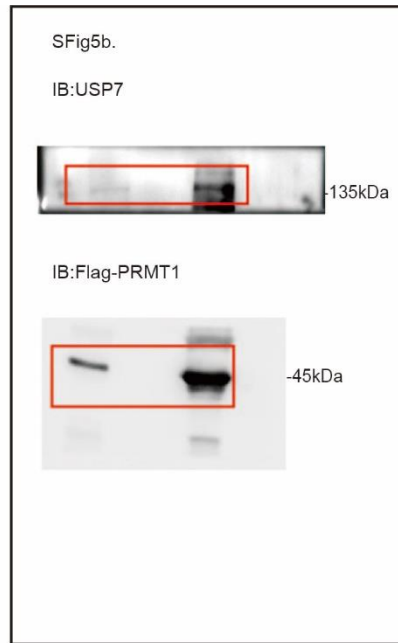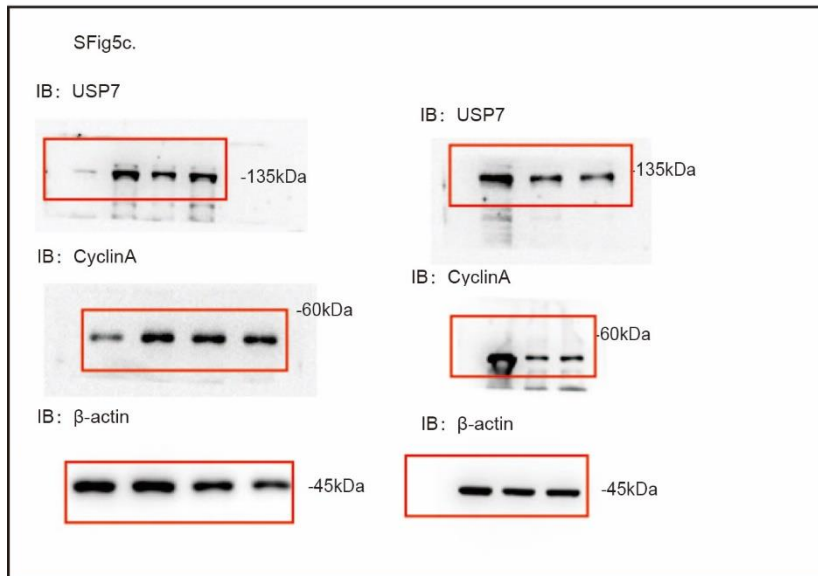

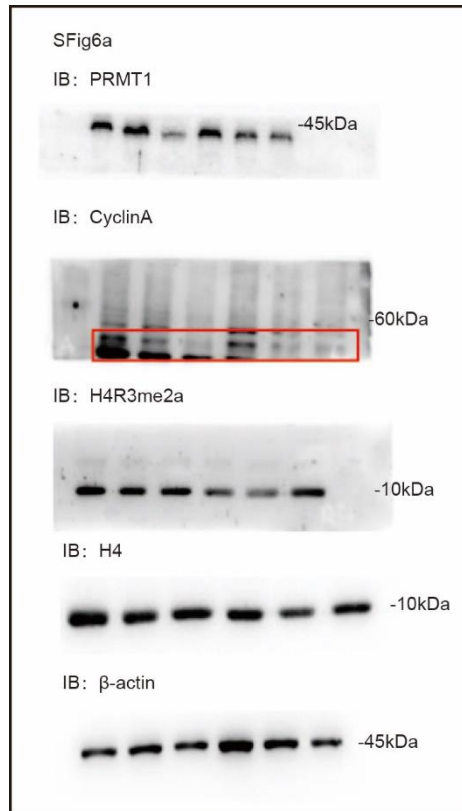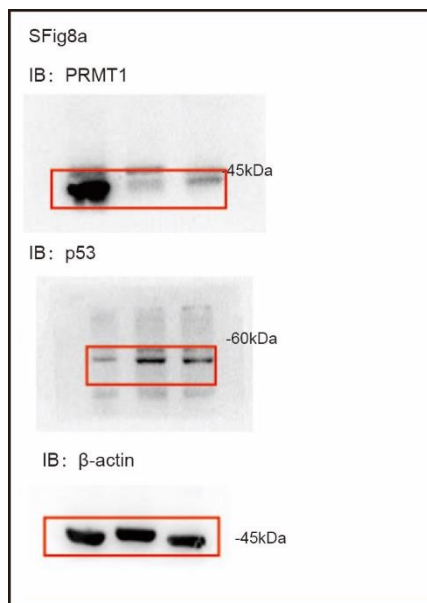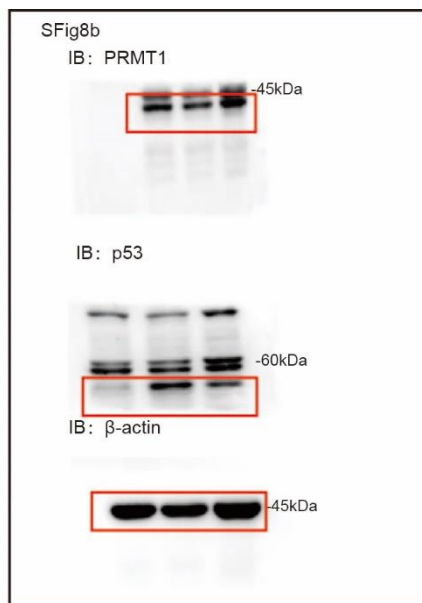

Supplement: Supplementary file 14 — Full and uncropped western blots [file 41419_2024_6898_MOESM14_ESM.pdf]
