# Supplementary material for: PRMT1 promotes Warburg effect by regulating the PKM2/PKM1 ratio in non-small cell lung cancer
Source: Cell Death Dis. 2024 Jul 15;15(7):504. doi: 10.1038/s41419-024-06898-x (PMC11251085; doi:10.1038/s41419-024-06898-x)
Supplement: Supplementary file 3 — Supplementary Materials and Methods [file 41419_2024_6898_MOESM3_ESM.docx]

**PRMT1 promotes Warburg effect by regulating the PKM2/PKM1 ratio in Non-small cell lung cancer**

**Supplementary Materials and Methods**

**Plasmids:**

The lentiviral expression plasmids pCDH-3×Flag-p53 R175H, pCDH-3×Flag-p53 R248Q, pCDH-3×Flag-p53 R273H were generated by standard cloning techniques using a two-step PCR procedure. Furthermore, shp53, shPRMT1 and shUSP7 were designed and cloned into another lentiviral RNAi system pLKO.1. The sequences of the shRNAs were listed as follows.

shp53#1:

5'-CCGGCGGCGCACAGAGGAAGAGAATCTCGAGATTCTCTTCCTCTGTGCGCCGTTTTTG-3' and 5'- AATTCAAAAACGGCGCACAGAGGAAGAGAATCTCGAGATTCTCTTCCTCTGTGCGCCG -3'

shp53#2:

5'- CCGGGTCCAGATGAAGCTCCCAGAACTCGAGTTCTGGGAGCTTCATCTGGACTTTTTG -3' and 5'- AATTCAAAAAGTCCAGATGAAGCTCCCAGAACTCGAGTTCTGGGAGCTTCATCTGGAC -3'

shPRMT1#1: 5'-CCGGGTGTTCCAGTATCTCTGATTACTCGAGTAATCAGAGATACTGGAACACTTTTTG -3' and 5'- AATTCAAAAAGTGTTCCAGTATCTCTGATTACTCGAGTAATCAGAGATACTGGAACAC -3'

shPRMT1#2: 5'- CCGGCCGGCAGTACAAAGACTACAACTCGAGTTGTAGTCTTTGTACTGCCGGTTTTTG -3' and 5'- AATTCAAAAACCGGCAGTACAAAGACTACAACTCGAGTTGTAGTCTTTGTACTGCCGG -3'

shUSP71#1: 5’-GATCCCCCCTGGATTTGTGGTTACGTTATTCAAGAGATAACGTAACCACAAATCCAGGTTTTTC-3’ and 5’-TCGAGAAAAACCTGGATTTGTGGTTACGTTATCTCTTGAATAACGTAACCACAAATCCAGGGG-3’

shUSP7#2: 5’-GATCCCCCGTGGTGTCAAGGTGTACTAATTCAAGAGATTAGTACACCTTGACACCACGTTTTTC-3' and 5’TCGAGAAAAACGTGGTGTCAAGGTGTACTAATCTCTTGAATTAGTACACCTTGACACCACGGGG-3’

**RNA extraction and qRT-PCR analysis**

Total RNA derived from NSCLC cells was extracted using TRIzol reagent (TAKARA, Dalian, China) according to the manufacturer’s instructions. RNA was reverse transcribed into cDNA using a Primer-Script one step RT-PCR kit (Monad, Changchun, China). Quantitative real-time PCR experiments were performed using a SYBR Premix Dimmer Eraser kit (TAKARA, Dalian, China). Relative mRNA expression was normalized to β-actin expression. The primer sequences used are shown in Supplementary Table 2.

**Extracellular acidification rate measurement**

The extracellular acidification rate (ECAR) was measured using a Seahorse XFp Extracellular Flux Analyzer (Seahorse Biosciences). Briefly, after cell transfection, 1×10^4^ cells/well was cultured in a XFp cell culture plate overnight. Cells were washed before incubation with assay medium (180μL) for 1 h at 37 °C without CO_2_. Then, cartridge ports A, B, and C were loaded with 20 μL of glucose (100 mM), 22 μL oligomycin (10 μM), and 25 μL 2-DG (500 M), respectively. The ECAR after glucose treatment indicated the glycolysis rate. The ECAR after oligomycin treatment indicated the glycolysis capacity.

**Oxygen consumption rate measurement**

The OCR was measured in an XF96 Extracellular Flux Analyzer (Seahorse Bioscience). Briefly, after cell transfection, 1×10^4^ cells/well was cultured in a XFp cell culture plate overnight. Cells were seeded into XFp Cell Culture Miniplates and incubated in a complete medium. The next day, the medium was changed to analysis media containing 2.0 mM L-glutamine, 10 mM glucose and 1.0 mM sodium pyruvate. The cells were incubated in a CO2-free incubator at 37 °C for 1 h. Cells were sequentially exposed to oligomycin (1 µM), FCCP (A549/H1299: 1 µM) and rotenone (1 µM).

**Supplementary Figure Legends**

**Supplement Figures:**

**Fig. S1.** **PRMT1 regulates PKM alternative splicing.** **A-B.** PRMT1 was knocked down in A549 and H1299 cells, and the exon 8 to exon 11 region of PKM was amplified by PCR, followed by detection of the relative amounts of PKM1 and PstI-digested PKM2.

**Fig. S2. Glucose promotes the level of PTBP1 and the PKM2/PKM1 ratio in NSCLC. A**. Different concentrations of glucose were treated with A549 for the indicated times, PKM1, PKM2, PTBP1 protein expression were detected by western blot. **B**. A549 was treated with melatonin at 2 mM for the indicated time.

**Fig. S3.** **PRMT1 increases PTBP1 transcription in NSCLC cells.** **A-B**. According to TCGA, PRMT1 and PTBP1 mRNA in lung adenocarcinoma samples were significantly higher than in normal lung tissue. **C**. Positive correlation between PRMT1 and PTBP1 gene expression in LUAD. **D**. HEK-293T cells were transfected with overexpressing PRMT1 at different concentrations, and the interaction between PRMT1 and the PTBP1 promoter was detected by the dual luciferase reporter gene. **E**. A549 cells were treated with different concentrations of the PRMT1 inhibitor AMI-1, and western blot analyses of PTBP1. H4R3me2a was used as a positive control. Error bars, mean ± SD. *P < 0.05; **P < 0.01; ***P < 0.001.

**Fig. S4. p53 inhibites the protein level of PRMT1 in NSCLC**. **A**. H1299 cells cultured in 0mM glucose for 0, 12, and 24 h were lysed, and following PRMT1 were detected by western blot. **B**. Overexpression of R175H, R248Q and R273H mutp53 in p53-null H1299 cells, PRMT1 expression in cells detected by Western-blot assays. **C-D**. Western blot analysis of PRMT1 in mutp53-transfected H1299 cells treated with glucose starvation and melatonin. **E**. Western blot and qPCR analyses of PRMT1 in A549-Vector, A549-p53 cells. **F**. Western blot and qPCR analyses of PRMT1 in A549-shCtrl, shp53#1, and shp53#2 cells. Error bars, mean ± SD. ns (non significant), P > 0.05, **P < 0.01; ****P < 0.0001.

**Fig. S5. USP7 binds to PRMT1 and the level of USP7 was correlated with the concentration of glucose.** **A**. The A549-Flag-PRMT1 cell line was cultured in high-glucose medium and low-glucose medium, respectively, for 24 h. The Flag-PRMT1 complex was isolated from the cells using affinity chromatography, and the proteins were recovered from the gel and analyzed by mass spectrometry. **B**. Western blot analyses of mutual co-immunoprecipitation of PRMT1 and USP7 in HEK-293T-PRMT1 cells. **C**. A549 cells were treated with different concentrations of glucose and melatonin for the indicated times, and western blot analyses of USP7 protein, CyclinA as a positive control.

**Fig. S6. PRMT1 inhibitor AMI-1 enhances the proliferation inhibitory effect of melatonin in p53-deficient NSCLC. A**. Western blot analyses of CyclinA in vitro AMI-1 (50μM) and melatonin(2mM) treatment of A549 cells. **B**. Cell proliferation rate by CCK8 in AMI-1 and melatonin treatment of A549 cells. **C**. AMI-1 and melatonin treatment of A549 cells and H1299 cells, monoclonal formation assay for cell proliferation levels. Error bars, mean ± SD. ns (non significant), P > 0.05, *P < 0.05; **P < 0.01; ***P < 0.001.

**Fig. S7. The body weight of the mice was not affected by the drug. A.** Nude mice were transplanted with A549 cells and treated with melatonin (20 mg/kg), and body weights were measured (5 mice/group). **B**. Nude mice were transplanted with H1299 cells and given melatonin (20 mg/kg), P5091 (10 mg/kg) or combined melatonin, and body weights were measured. (5 mice/group). Error bars, mean ± SD. ns (non significant), P > 0.05.

**Fig. S8.** **PRMT1 inhibites p53 protein in NSCLC.** **A.** Western blot and qPCR analysis of p53 protein and mRNA in A549-shCtrl, shPRMT1#1, and shPRMT1#2 cells. **B.** Western blot analysis of PRMT1 and p53 protein levels in A549-p53 cells overexpressing PRMT1. Error bars, mean ± SD. ns (non significant), P > 0.05.

**Fig. S9. PRMT1 and USP7 was highly expressed in NSCLC and predicted poor prognosis.** **A**. The expression of PRMT1 and USP7 gene expression in different types of cancer was analyzed by the TIMER2.0 network tool. Tumor (red); Normal tissue (blue). LUAD, lung adenocarcinoma; LUSC, lung squamous cell carcinoma. **B**. The expression of PRMT1 and USP7 gene expression in different types of NSCLC was analyzed by the UALCAN network tool. TP53-Mutant (red); TP53-NonMutant (yellow); Normal tissue (blue). **C.** Survival of 481 PRMT1 and USP7 high-expressing (red), 481 PRMT1 and USP7 low-expressing (blue) patients were analyzed by the GEPIA network tool.
