# Supplementary material for: PRMT1 promotes Warburg effect by regulating the PKM2/PKM1 ratio in non-small cell lung cancer
Source: Cell Death Dis. 2024 Jul 15;15(7):504. doi: 10.1038/s41419-024-06898-x (PMC11251085; doi:10.1038/s41419-024-06898-x)
Supplement: Supplementary file 4 — Supplementary Table 1 [file 41419_2024_6898_MOESM4_ESM.docx]

**Supplementary Table 1 Reagents**

| **Reagents** | **Catalog Number** |
| --- | --- |
| Pst1 | NEB D6565 |
| Melatonin | Beyotime ST1497 |
| Pifithrin-α | Beyotime S1816 |
| P5091 | Selleck S7132 |
| RNase | Roche Applied Science |
| Proteinase K | Invitrogen 25530015 |
| Glucose | Sigma G7021 |
| Oligomycin | Abcam ab141829 |
| 2-DG | Beyotime ST1024 |
| Glutamine | Beyotime ST083 |
| FCCP | Abcam ab120081 |
| Rotenone | Abcam ab143145 |
| Pyruvate Kinase(PK) Activity Assay Kit | Solarbio BC0545 |
| ATP Assay Kit | Beyotime, S0026 |
| Lactate Assay Kit | sigma-aldrich MAK064 |
| L-lactic acid (L-LA) content detection kit | Solarbio BC2230 |
| CHIP | CST #9006 |

**Supplementary** **Table 1 Antibody**

| **Antibody** | **Catalog Number** |
| --- | --- |
| PRMT1 | ABclonal A1055 |
| PRMT1（CO-IP） | CST A33 |
| p53 | Sigma P6749 |
| p21 | Proteintech 10355 |
| β-actin | Sigma A1978 |
| cyclin A2 | Abcam ab181591 |
| LC3BII | CST 3868 |
| USP7 | Abways CY7219 |
| USP9X | Proteintech 55054-1-AP |
| Flag | Abmart M20008 |
| HIS | Abmart M30111 |
| Ubiquitin | CST #3936 |
| PKM1 | ABclonal A21052 |
| PKM2 | ABclonal A20991 |
| HnRNPA1 | ABclonal A11564 |
| HnRNPA2B1 | ABclonal A21802 |
| PTBP1 | Abclonal A3487 |
| LaminB1 | ABclonal A1910 |
| CARM1 | Millipore P4995 |
| PRMT6 | CST #14641 |
