# Supplementary material for: PRMT1 promotes Warburg effect by regulating the PKM2/PKM1 ratio in non-small cell lung cancer
Source: Cell Death Dis. 2024 Jul 15;15(7):504. doi: 10.1038/s41419-024-06898-x (PMC11251085; doi:10.1038/s41419-024-06898-x)
Supplement: Supplementary file 5 — Supplementary Table 2 [file 41419_2024_6898_MOESM5_ESM.docx]

**Supplementary Table 2 RT-PCR Primers**

|  |  |
| --- | --- |
| PRMT1 sense | 5'-AGCGAAGTGCCAACACCTAAG-3' |
| PRMT1 antisense | 5'-TGGTGGTTTTCCGGGTCTTG-3' |
| p53 sense | 5'-ACCCAGGGGAAGACCCAAA-3' |
| P53 antisense | 5'-CCTCTTGGCCGTTTTTCTCCA-3' |
| PTBP1 sense | 5'-TTCATTTAGCACTCTACACAGTCACGG-3' |
| PTBP1 antisense | 5'-TTGAGGTAGGTCTGGTGAAGGTCC-3' |
| PKM1 sense | 5'-TGGAAAAGTTGGCTCCAAAG-3' |
| PKM1 antisense | 5'-TCAGAAAAAGCTTGGCAGAGA-3' |
| PKM2 sense | 5'-CCCTTTAGCGCGGATCTACC-3' |
| PKM2 antisense | 5'-CATGGCTACCACTTGACCTGT-3' |
| Glut1 sense | 5'-TGTTCTCCTCGTGTAAAAGCC-3' |
| Glut1 antisense | 5'-GAATGGCAGGCTTGGTGATG-3' |
| Glut4 sense | 5'-ACCGTCACTATGGACCAGC-3' |
| Glut4 antisense | 5'-TTCAGAGCTGGACTACATCC-3' |
| PKFB3 sense | 5'-CGATTGGCGCGAAGTTTTCT-3' |
| PKFB3 antisense | 5'-CTTGTGGGGTAGTGTGCCAT-3' |
| PGC-1α sense | 5'-CGTGACCTACATCGACGAGA-3' |
| PGC-1α antisense | 5'-GGAGGGCAGCTATTAGGAGG-3' |
| HK2 sense | 5'-TGTCACCTGCTCTGCCACTAA-3' |
| HK2 antisense | 5'-GCAGCATGCGCAAGACTTT-3' |
| LDH sense | 5'-CAGTGGCCATCCATCCTGTT-3' |
| LDH antisense | 5'-TCGACAGGAGCTGCATGTTT-3' |
| ENO1 sense | 5'-CAGACTACGAGGCGTCATCC-3' |
| ENO1 antisense | 5'-TCTGCGGGTGAGTGGTAGTA-3' |
| cyclinA sense | 5'-GAGCTGGAGCGCCTGATAAT-3' |
| cyclinA antisense | 5'-CCCTCCTGCTCATCTGTCAC-3' |
