# Supplementary material for: PRMT1 promotes Warburg effect by regulating the PKM2/PKM1 ratio in non-small cell lung cancer
Source: Cell Death Dis. 2024 Jul 15;15(7):504. doi: 10.1038/s41419-024-06898-x (PMC11251085; doi:10.1038/s41419-024-06898-x)
Supplement: Supplementary file 6 — Supplementary Table 3 [file 41419_2024_6898_MOESM6_ESM.docx]

**Supplemental table 3. The potential interaction proteins of PRMT1.**

| **Accession** | **Description** | **MW**  **[kDa]** | **calc. pI** |
| --- | --- | --- | --- |
| P35579 | Myosin-9  (MYH9) | 226.4 | 5.6 |
| P35580 | Myosin-10 (MYH10) | 228.9 | 5.54 |
| **A0A3S6H812** | **Protein arginine N-methyltransferase 1 (PRMT1)** | **43.8** | **5.99** |
| **H3BND8** | **Ubiquitin carboxyl-terminal hydrolase (Fragment) (USP7)** | **105.5** | **5.58** |
| **Q6P468** | **USP9X protein (Fragment) (USP9X)** | **105.8** | **6.23** |
| **F8W6I7** | **Helix-destabilizing protein (HNRNPA1)** | **33.1** | **9.13** |
| **D6RBZ0** | **Heterogeneous nuclear ribonucleoprotein A/B (HNRNPAB)** | **35.7** | **6.95** |
| A0A024RCT9 | High mobility group AT-hook protein 1 (HMGA1) | 34.3 | 10.37 |
